# SARS-CoV-2 Diverges from Other Betacoronaviruses in Only Partially Activating the IRE1α/XBP1 Endoplasmic Reticulum Stress Pathway in Human Lung-Derived Cells

**DOI:** 10.1128/mbio.02415-22

**Published:** 2022-09-20

**Authors:** Long C. Nguyen, David M. Renner, Diane Silva, Dongbo Yang, Nicholas A. Parenti, Kaeri M. Medina, Vlad Nicolaescu, Haley Gula, Nir Drayman, Andrea Valdespino, Adil Mohamed, Christopher Dann, Kristin Wannemo, Lydia Robinson-Mailman, Alan Gonzalez, Letícia Stock, Mengrui Cao, Zeyu Qiao, Raymond E. Moellering, Savas Tay, Glenn Randall, Michael F. Beers, Marsha Rich Rosner, Scott A. Oakes, Susan R. Weiss

**Affiliations:** a Ben May Department for Cancer Research, University of Chicago, Chicago, Illinois, USA; b Department of Pathology, University of Chicago, Chicago, Illinois, USA; c Department of Microbiology, University of Chicago, Chicago, Illinois, USA; d Pritzker School of Molecular Engineering, University of Chicago, Chicago, Illinois, USA; e Department of Chemistry, University of Chicago, Chicago, Illinois, USA; f Department of Microbiology, University of Pennsylvania, Philadelphia, Pennsylvania, USA; g Department of Medicine, University of Pennsylvania, Philadelphia, Pennsylvania, USA; h Penn-CHOP Lung Biology Institute, University of Pennsylvania, Philadelphia, Pennsylvania, USA; i Penn Center for Research on Coronaviruses and Other Emerging Pathogens, Perelman School of Medicine, University of Pennsylvania, Philadelphia, Pennsylvania, USA; j Howard Taylor Ricketts Laboratory, Argonne National Laboratory, Lemont, Illinois, USA; Johns Hopkins Bloomberg School of Public Health

**Keywords:** IRE1α pathway, MERS-CoV, OC43, SARS-CoV-2, coronavirus, unfolded protein response

## Abstract

Severe acute respiratory syndrome coronavirus 2 (SARS-CoV-2) has killed over 6 million individuals worldwide and continues to spread in countries where vaccines are not yet widely available or its citizens are hesitant to become vaccinated. Therefore, it is critical to unravel the molecular mechanisms that allow SARS-CoV-2 and other coronaviruses to infect and overtake the host machinery of human cells. Coronavirus replication triggers endoplasmic reticulum (ER) stress and activation of the unfolded protein response (UPR), a key host cell pathway widely believed to be essential for viral replication. We examined the master UPR sensor IRE1α kinase/RNase and its downstream transcription factor effector XBP1s, which is processed through an IRE1α-mediated mRNA splicing event, in human lung-derived cells infected with betacoronaviruses. We found that human respiratory coronavirus OC43 (HCoV-OC43), Middle East respiratory syndrome coronavirus (MERS-CoV), and murine coronavirus (MHV) all induce ER stress and strongly trigger the kinase and RNase activities of IRE1α as well as XBP1 splicing. In contrast, SARS-CoV-2 only partially activates IRE1α through autophosphorylation, but its RNase activity fails to splice XBP1. Moreover, while IRE1α was dispensable for replication in human cells for all coronaviruses tested, it was required for maximal expression of genes associated with several key cellular functions, including the interferon signaling pathway, during SARS-CoV-2 infection. Our data suggest that SARS-CoV-2 actively inhibits the RNase of autophosphorylated IRE1α, perhaps as a strategy to eliminate detection by the host immune system.

## INTRODUCTION

**FIG 1:**
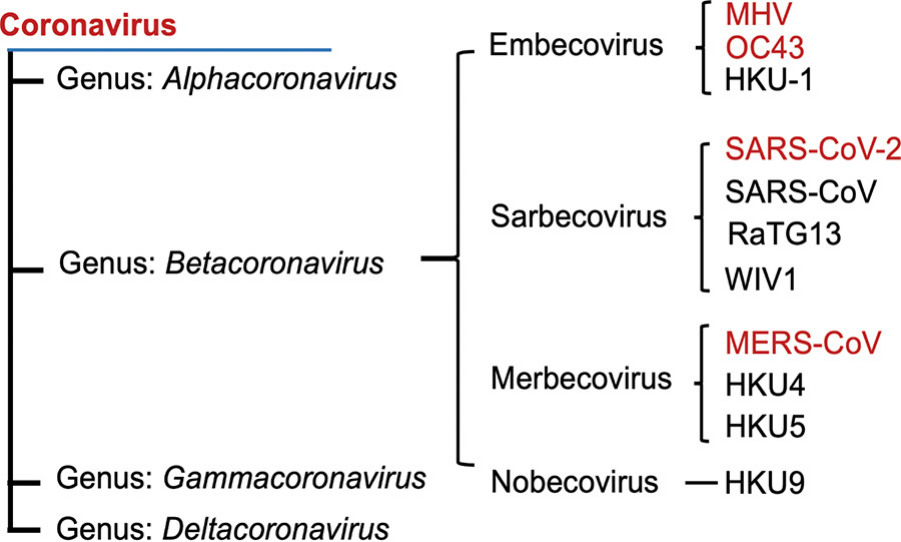
Coronavirus family. Phylogenetic tree of betacoronaviruses and their subgenera. Viruses examined in this study are shown in red font.

Severe acute respiratory syndrome coronavirus 2 (SARS-CoV-2) emerged in China in late 2019. It was the third lethal zoonotic coronavirus to emerge into humans, after SARS-CoV (2002) and Middle East respiratory syndrome coronavirus (MERS-CoV) (2012), each of which has been associated with acute lung injury and hypoxemic respiratory failure. While coronaviruses are divided into four genera (alpha, beta, gamma, and delta) (1, 2), all three of the lethal human coronaviruses are betacoronaviruses, albeit from different subgenera (Fig. 1). SARS-CoV and SARS-CoV-2 are sarbecoviruses, while MERS-CoV is a merbecovirus. Other human CoVs, including HCoV-OC43 (OC43) and HCoV-HKU1 (HKU-1), are embecoviruses, as is the model murine coronavirus mouse hepatitis virus (MHV). All CoVs have similar genome structures and replication cycles, and the human CoVs as well as some MHV strains exhibit tropism for the epithelia of the respiratory tract, the portal of entry. They replicate their RNAs and produce subgenomic mRNAs by conserved mechanisms and encode homologous structural as well as replicase proteins. Despite the similarities among all coronaviruses, each subgenus expresses distinct accessory proteins that may confer differences in host-virus interactions. Indeed, we have previously found that SARS-CoV-2, MERS-CoV, and MHV all induce somewhat different levels of activation and/or antagonism of interferon (IFN) signaling and other double-stranded RNA (dsRNA) induced antiviral innate responses (3–11).

One key pathway involved in the virus-induced host response is the endoplasmic reticulum (ER) stress response that regulates protein homeostasis (referred to as proteostasis) in this organelle. One-third of all eukaryotic proteins, including most that are inserted into membranes or secreted, are synthesized through co-translational translocation into the ER lumen. Likewise, viral membrane-associated proteins are translated and processed in association with the ER (12, 13). Once in the ER, these polypeptides undergo stringent quality control monitoring to ensure that they are properly processed and folded. If the capacity to fold proteins is unable to keep up with demand, misfolded proteins will accumulate in the ER lumen—a condition referred to as “ER stress.” The presence of misfolded proteins in the ER is sensed by three transmembrane sentinel proteins—activating transcription factor 6 (ATF6), PKR-like ER kinase (PERK), and inositol-requiring enzyme (IRE)1α—which trigger an intracellular signaling pathway called the unfolded protein response (UPR). In an effort to restore proteostasis, activation of these sensors induces transcription factors that turn on genes encoding chaperones, oxidoreductases, and ER-associated decay (ERAD) components (14). The UPR also inhibits cap-dependent translation, thus decreasing the load on the ER and giving it extra time to fold proteins already in production (15, 16). If successful, these adaptive UPR programs restore ER homeostasis.

The most ancient UPR pathway is controlled by IRE1α—an ER transmembrane bifunctional kinase/endoribonuclease (RNase) that employs autophosphorylation to control its catalytic RNase function (17, 18). In response to ER stress, IRE1α undergoes autophosphorylation and dimerization to allosterically activate its RNase domain to excise a 26-nucleotide (nt) nonconventional intron in *XBP1* mRNA; religation of spliced *XBP1* shifts the open reading frame, and its translation produces the homeostatic transcription factor XBP1s (s = spliced) (19, 20). Once synthesized, XBP1s upregulates genes that expand the ER and its protein folding machinery (21). IRE1α can additionally lead to apoptosis and inflammation via JUN N-terminal kinase (JNK) and p38 mitogen-activated protein kinase (MAPK) signaling (22). Prolonged ER stress can induce regulated IRE1-dependent decay (RIDD), promoting the cleavage of additional targets beyond XBP1 mRNA, such as secretory protein and ER-localized mRNAs (23). In the short term, RIDD may promote adaptation through further reducing translation and the protein burden on the ER. However, prolonged RIDD leads to the depletion of vital ER resident enzymes and structural components to exacerbate ER stress and hasten cell death (17, 24).

There is a large body of evidence that viral replication in mammalian cells can trigger ER stress and UPR activation in infected cells (25), and numerous studies report that the UPR is activated upon infection of host cells by coronavirus family members (12, 13, 26–31). Coronaviruses induce stress in the ER in several ways. First, conserved replicase-encoded, nonstructural proteins nsp3, nsp4, and nps6 are embedded into the ER membrane and, along with unknown host factors, promote membrane curvature to form double membrane vesicles (DMVs), the site of viral replication/transcription centers (RTC) (32). In addition to remodeling the ER, coronaviruses further condition infected cells by shifting translation away from host mRNAs and instead to viral mRNAs. Translation of viral mRNAs causes the ER to be flooded with heavily glycosylated viral structural proteins (e.g., spike [S], membrane [M], and envelope [E]), challenging the organelle’s folding capacity and overall integrity. Indeed, overexpression of coronavirus spike proteins (33) as well as several sarbecovirus accessory proteins (28, 34), has been reported to induce ER stress, although overexpression itself may cause stress irrespective of the proteins. Finally, cell membranes are depleted as enveloped virus particles are assembled into new virions in the ER-Golgi intermediate compartment before budding from the infected cell (1). Thus, coronaviruses as well as other enveloped viruses promote a massive ER expansion and modification necessary to replicate their genomes, transcribe mRNAs, and finally, to process and package their protein products into viral particles.

We have compared the activation status and requirement of the IRE1α/XBP1 arm of the UPR in well-characterized human lung epithelial cell lines and in induced pluripotent stem cell (iPSC)-derived type II alveolar (iAT2) cells, following infection with four betacoronaviruses representing three distinct subgenera. We find that infection with MERS-CoV, OC43, and MHV leads to phosphorylation of IRE1α and the consequent production of spliced XBP1 (XBP1s) transcription factor. Surprisingly, while we observed phosphorylation of IRE1α in SARS-CoV-2 infected cells, there was a notable absence of XBP1s, suggesting that SARS-CoV-2 inhibits downstream signaling of the IRE1α/XBP1 arm of the UPR. In addition, we report reduced SARS-CoV-2-induced interferon signaling gene expression in the absence of IRE1α.

## RESULTS

### Induction of IRE1α phosphorylation following coronavirus infection.

**FIG 2:**
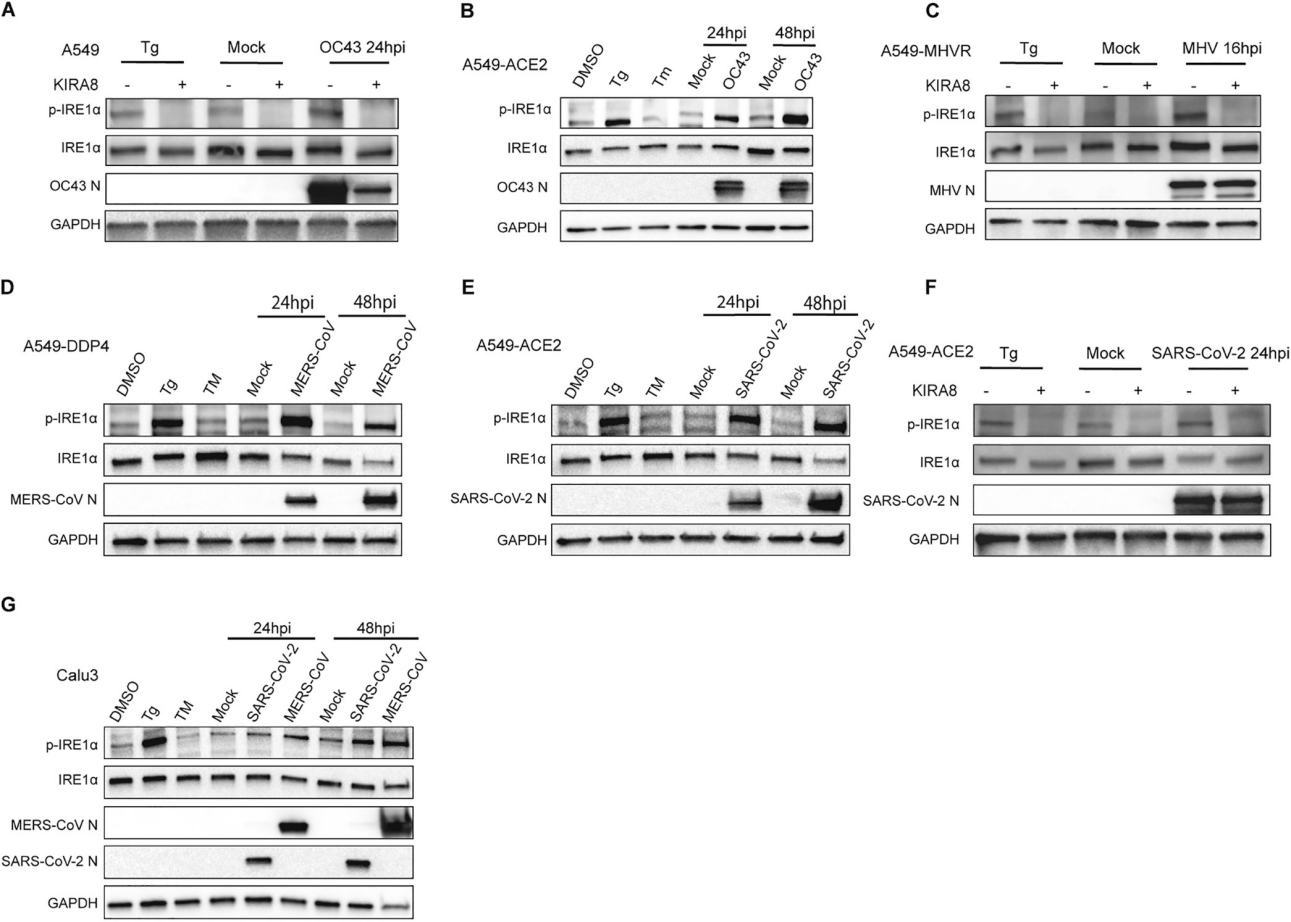
Induction of IRE1α phosphorylation following coronavirus infection. A549 cells expressing the indicated viral receptors were mock infected or infected. Protein was harvested at 16, 24, or 48 hpi and analyzed by immunoblotting with antibodies, as indicated. (A, C, and F) Cells infected with OC43 (A), MHV (C), or SARS-CoV-2 (F) at an MOI of 5 were pretreated 2 h prior to infection with 1 μM KIRA8. (B, D, and E) Cells were infected with OC43 at an MOI of 1 (B), MERS-CoV at an MOI of 5 (D), or SARS-CoV-2 at an MOI of 5 (E) or treated with DMSO, thapsigargin (Tg; 1 μM) for 1 h or tunicamycin (TM, 1 μg/mL) for 8 h. (G) Calu-3 cells were mock infected or infected with MERS-CoV or SARS-CoV-2 (MOI, 5). Data shown are from one representative of at least two independent experiments.

To determine whether betacoronaviruses activate IRE1α, we first examined the level of phosphorylated IRE1α after viral infection of the A549 human lung carcinoma cell line. We used A549 cells stably expressing the following receptors to facilitate optimal entry for each of the viruses: carcinoembryonic antigen cell adhesion molecule (CEACAM) 1a or MHVR (MHV), dipeptidyl peptidase DPP4 (MERS-CoV) or angiotensin-converting enzyme 2 (ACE2) (SARS-CoV-2). HCoV-OC43 can infect parental A549 or cells expressing ACE2 (3). Consistent with previous reports that embeco subgenus coronaviruses MHV (26, 35) and OC43 (30) induce ER stress, we observed a significant increase in phospho-IRE1α (p-IRE1α) during infection by either OC43 (24 or 48 h postinfection [hpi]) or MHV (24 hpi) (Fig. 2A to C). To confirm the specificity of the p-IRE1α band, we pretreated cells prior to infection with KIRA8, a highly selective kinase inhibitor of IRE1α known to inhibit both autophosphorylation and, consequently, RNase activity. As expected, KIRA8 significantly inhibited the induction of p-IRE1α by OC43 and MHV (Fig. 2A and C). Thapsigargin (Tg) and tunicamycin (TM), both inducers of ER stress, were used as further controls (Fig. 2B and D, and E). Robust induction of p-IRE1α was observed with 1 h of Tg (1 μM) treatment, while no activation of p-IRE1α was observed after 8 h of treatment with TM (1 μg/mL), consistent with the negative feedback regulation observed with extended TM treatment (36). We also observed robust phosphorylation of IRE1α in A549-DDP4 cells and A549-ACE2 cells infected by MERS-CoV and SARS-CoV-2, respectively, at 24 and 48 hpi (Fig. 2D to F and Fig. S1A and B in the supplemental material). As with OC43 and MHV, IRE1α phosphorylation during SARS-CoV-2 infection was inhibited by KIRA8 (Fig. 2F). Interestingly, we observed a decrease in OC43 (Fig. 2A) and SARS-CoV-2 (Fig. 2F) nucleocapsid expression in KIRA8-treated cells. However, this may be the result of off-target effects from the compound rather than solely from IRE1α inhibition, given our findings described below using IRE1α knockout (KO) cells. These results are not limited to a single cell type, as we observed similar induction of p-IRE1α in Calu-3 cells, another lung epithelial-derived cells line, which can be productively infected with both MERS-CoV or SARS-CoV-2 (Fig. 2G). These results demonstrate that MERS-CoV, SARS-CoV-2, HCoV-OC43, and MHV activate the host IRE1α kinase after infection.

10.1128/mbio.02415-22.1 FIG S1 Kinetics of activation of IRE1α phosphorylation during infection with MERS-CoV or SARS-CoV-2. (A and B) A549 cells expressing the indicated viral receptors were mock infected or infected with MERS-CoV (A) or SARS-CoV-2 (B) at an MOI of 5. At the indicated time points, total protein was harvested and analyzed by immunoblotting with the indicated antibodies. Cells treated with thapsigargin (Tg; 1μM) for 1 h or tunicamycin (TM; 1 μg/mL) for 8 h were used as a positive control for IRE1α phosphorylation and attenuation, respectively. Data shown are from one representative experiment from at least two independent experiments. Download FIG S1, PDF file, 0.3 MB. Copyright © 2022 Nguyen et al. 2022 Nguyen et al. https://creativecommons.org/licenses/by/4.0/ This content is distributed under the terms of the Creative Commons Attribution 4.0 International license.

### MHV, OC43, and MERS-CoV but not SARS-CoV-2 induce splicing of XBP1 mRNA.

**FIG 3:**
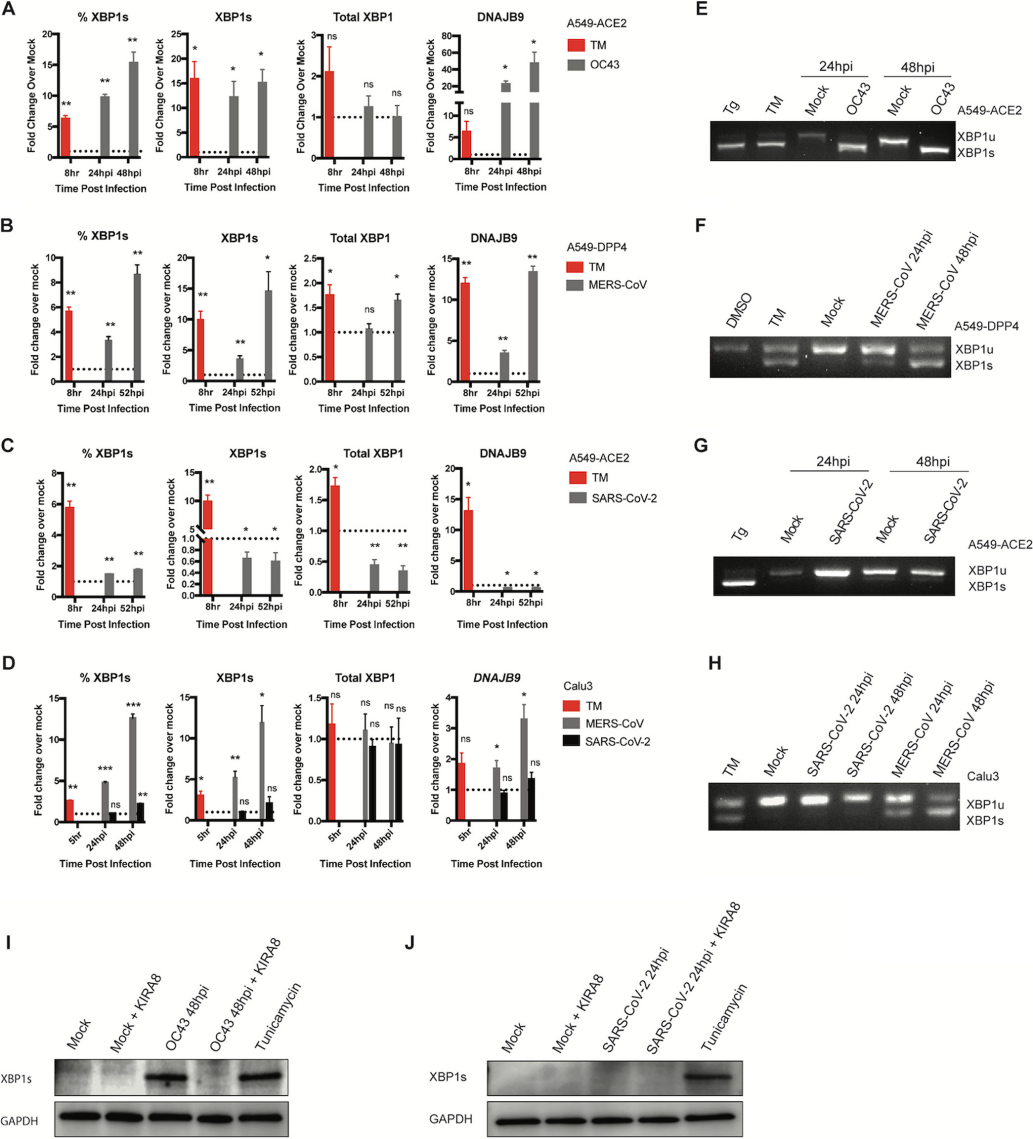
IRE1α-mediated XBP1 splicing occurs following infection with OC43 or MERS-CoV, but not SARS-CoV-2. (A to C, E to G) A549 cells were mock infected or infected (in triplicate) with OC43 at an MOI of 1 (A, E), MERS-CoV at an MOI of 5 (B, F), or SARS-CoV-2 at an MOI of 5 (C, G) or treated with TM (1 μg/mL) for 8 h, and total RNA was harvested at the indicated time points. (A to C) Relative %XBP1s, XBP1s, total XBP1, and DNAJB9 mRNA expression were quantified by RT-qPCR. *C_T_* values were normalized to 18S rRNA and expressed as the fold change over the mock control displayed as 2^−Δ(Δ^*^CT^*^)^. Technical replicates were averaged, and the means for each replicate were displayed ± the standard deviation (SD; error bars). (D) Calu-3 cells were mock infected or infected with MERS-CoV or SARS-CoV-2 (MOI, 5) and total RNA was harvested at the indicated time points. Relative %XBP1s, XBP1s and total XBP1 and DNAJB9 mRNA expression were quantified by RT-qPCR, calculated, and displayed as described above. Values are means ± SD (error bars). Statistical significance was determined using two-tailed, paired Student’s *t* test. Displayed significance (infected relative to mock) is determined by the *P* value; *, *P* < 0.05; **, *P* < 0.01; ***, *P* < 0.001; ****, *P* < 0.0001; ns, not significant. (E to H) RNA was harvested from A549 cells mock infected or infected with OC43 at an MOI of 1 (E), MERS-CoV at an MOI of 5 (F), SARS-CoV-2 at an MOI of 5 (G), or Calu-3 cells infected with MERS-CoV and SARS-CoV-2 at an MOI of 5 (H) or treated with tunicamycin (TM; 1 μg/mL) for 8 h, or thapsigargin (Tg; 1 μM) for 1 h or DMSO. RT-PCR was performed using primers crossing the XBP1 splicing site. The product was resolved on an agarose gel to visualize XBP1 splicing. (I to J) Lysates from A549-ACE2 cells mock infected, treated with TM (500 ng/mL) for 6 h, or infected with OC43 (MOI, 4) or SARS-CoV-2 (MOI, 3) with or without KIRA8 (1 μM) treatment were harvested at the indicated time points as in Fig. 2A, C and F and immunoblotted with antibody against XBP1s protein. Data shown are from one representative experiment from at least three independent experiments.

We next examined the effect of coronavirus infection on the RNase activity of IRE1α as assessed by XBP1 splicing. Using specific primers to quantify spliced XBP1 mRNA (XBP1s), we observed a marked increase in the percentage of spliced XBP1 mRNA (%XBP1s) as well as an increase in the relative amount of spliced XBP1 mRNA (XBP1s) compared to the mock control after infection by OC43, MERS-CoV, or MHV in receptor-expressing A549 cells (Fig. 3A and B and Fig. S2A and B). This induction of XBP1s by OC43 and by MERS-CoV infection was confirmed by assessing XBP1 splicing by agarose gel electrophoresis (Fig. 3E and F). DNAJB9, a canonical target of XBP1s, was also markedly upregulated with OC43, MERS-CoV, and MHV infection at both 24 and 48 hpi (Fig. 3A and B and Fig. S2B). This induction of IRE1α RNase activity is coincident with the observed autophosphorylation of p-IRE1α upon OC43, MHV, or MERS-CoV infection.

10.1128/mbio.02415-22.2 FIG S2 XBP1 is spliced in MHV-infected cells. (A) Schematic of method and primer design used to quantify %XBP1. (B) A549-MHVR cells were mock infected or infected with MHV (MOI, 0.1). Total RNA was harvested at 48 h postinfection. Relative %XBP1s, XBP1s, total XBP1, and DNAJB9 mRNA expression were quantified by RT-qPCR. *C_T_* values were normalized to 18S rRNA and expressed as the fold change over the mock control displayed as 2^−Δ(Δ^*^CT^*^)^. Technical replicates were averaged, and the mean for each biological replicate (*n* = 2) is displayed ± SD (error bars). Download FIG S2, PDF file, 0.05 MB. Copyright © 2022 Nguyen et al. 2022 Nguyen et al. https://creativecommons.org/licenses/by/4.0/ This content is distributed under the terms of the Creative Commons Attribution 4.0 International license.

Surprisingly, despite the observed IRE1α autophosphorylation following SARS-CoV-2 infection, there was no significant upregulation of XBP1s mRNA in A549-ACE2 cells up to 52 hpi (Fig. 3C and G). Similarly, DNAJB9 expression levels were unchanged at all time points observed with SARS-CoV-2 (Fig. 3C). To confirm that this effect is not limited to A549 cells, we measured XBP1 mRNA splicing in MERS-CoV- and SARS-CoV-2-infected Calu-3 cells. Again, infection with MERS-CoV, but not SARS-CoV-2, significantly induced XBP1s and its downstream effector DNAJB9 (Fig. 3D and H). In agreement with these results, OC43, but not SARS-CoV-2, infection induced XBP1s protein levels (Fig. 3I and J).

### Upon infection, MHV, OC43, and MERS-CoV induce IRE1α and related genes to a greater extent than SARS-CoV-2.

**FIG 4:**
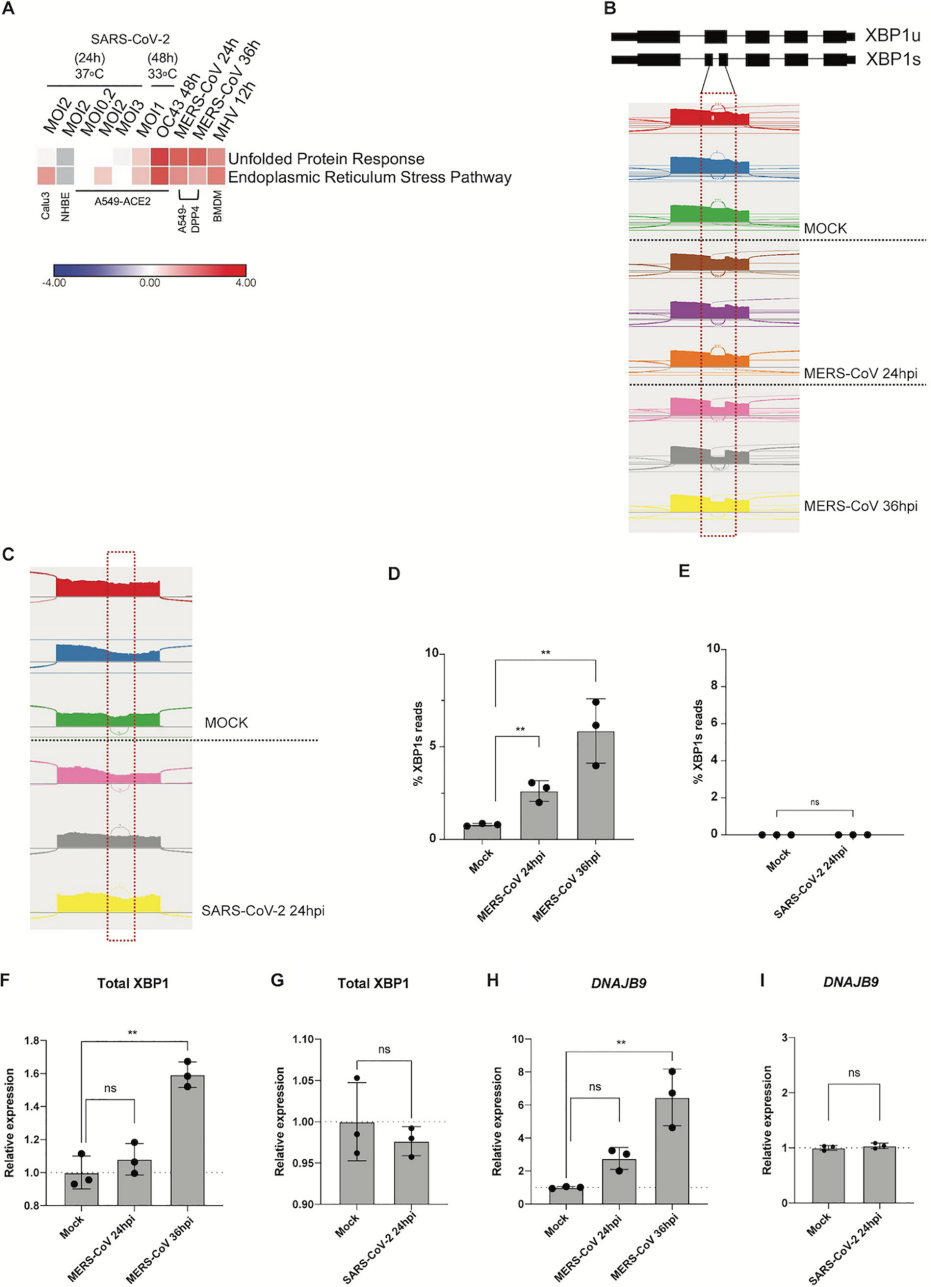
Unlike other coronaviruses, SARS-CoV-2 infection does not lead to robust UPR activation. (A) Heatmap of predicted pathway status based on Ingenuity Pathway Analysis (IPA) of activation Z-scores for each pathway from RNA-sequencing data from the indicated cells infected with OC43 (MOI, 1), MERS-CoV (MOI, 1), MHV (MOI, 1), and SARS-CoV-2 under the specified conditions. Red, pathway predicted to be activated; blue, pathway predicted to be inhibited; white, pathway predicted to be unchanged; gray, no prediction due to lack of significance. (B and C) Quantification of XBP1 splicing by analyzing RNA-seq data from A549-DPP4 and A549-ACE2 cells mock infected or infected with MERS-CoV or SARS-CoV-2, respectively, under the indicated conditions. Reads representing spliced or unspliced XBP1 mRNA were identified based on the presence or absence of the 26-nucleotide intron and quantified. (D to I) Percentage of XBP1 spliced reads or relative expression of total XBP1 and DNAJB9 mRNA from the RNA-seq samples. Values are means ± SD (error bars). Statistical significance was determined by unpaired *t* tests (*, *P* < 0.05; **, *P* < 0.01; ns, not significant).

To determine how different coronaviruses impact the UPR at the transcriptional level, we performed RNA-sequencing of A549-DPP4 cells infected with MERS-CoV for 24 and 36 h. We compared the results to published RNA sequencing (RNA-seq) data sets (35, 37) of MHV infection of murine bone marrow-derived macrophages (BMDM) or SARS-CoV-2 infection of A549-ACE2, normal human bronchial epithelial (NHBE) cells, and Calu-3 cell lines. In agreement with our IRE1α activation results, Ingenuity Pathway Analysis (IPA) predicted activation of the UPR and ER stress pathways by MERS-CoV and MHV (Fig. 4A). In contrast, SARS-CoV-2 consistently showed little to no activation of the UPR and ER stress pathway across different multiplicity of infection (MOI) conditions and cell lines.

To confirm the results of the gel electrophoresis splicing assays for XBP1 mRNA that distinguished SARS-CoV-2 infection from that of the other betacoronaviruses (Fig. 3), we further utilized the RNA-seq results to quantitatively measure XBP1 mRNA splicing by these coronaviruses. Through RNA-seq, we visualized both the unspliced and spliced XBP1 mRNA reads based on whether they contain the 26-nucleotide nonconventional intron that is removed as a result of RNase activity of IRE1α as previously described (38) (Fig. 4B and C). MERS-CoV infection resulted in significant XBP1 mRNA splicing, in contrast to no difference detected in SARS-CoV-2-infected versus mock-infected cells (Fig. 4B and C). We further quantified total XBP1 spliced versus unspliced reads, which consistently showed a substantial increase in the percent expression of the XBP1s reads when normalized to total XBP1 reads for MERS-CoV at both 24 and 36 hpi but not for SARS-CoV-2-infected cells (Fig. 4D and E). This was consistent with significant upregulation of DNAJB9 and total XBP1 during infection with MERS-CoV but not SARS-CoV-2 (Fig. 4F to I).

### MERS-CoV but not SARS-CoV-2 induces XBP1 splicing during infection of biologically relevant iPSC-derived alveolar type II cells.

To confirm our results in a more physiologically relevant cell, we infected iPSC-derived type II alveolar (iAT2) cells. We employed the SPC2 line, which expresses tdTomato from the surfactant protein-C (SFTPC) locus as an AT2 marker, which we have previously used to characterize innate immune responses to SARS-CoV-2 infection (3). Type II alveolar cells are a major target during both MERS-CoV and SARS-CoV-2 infection in humans, and their destruction may be a contributing factor to lung pathogenesis in severe cases (39, 40).

**FIG 5:**
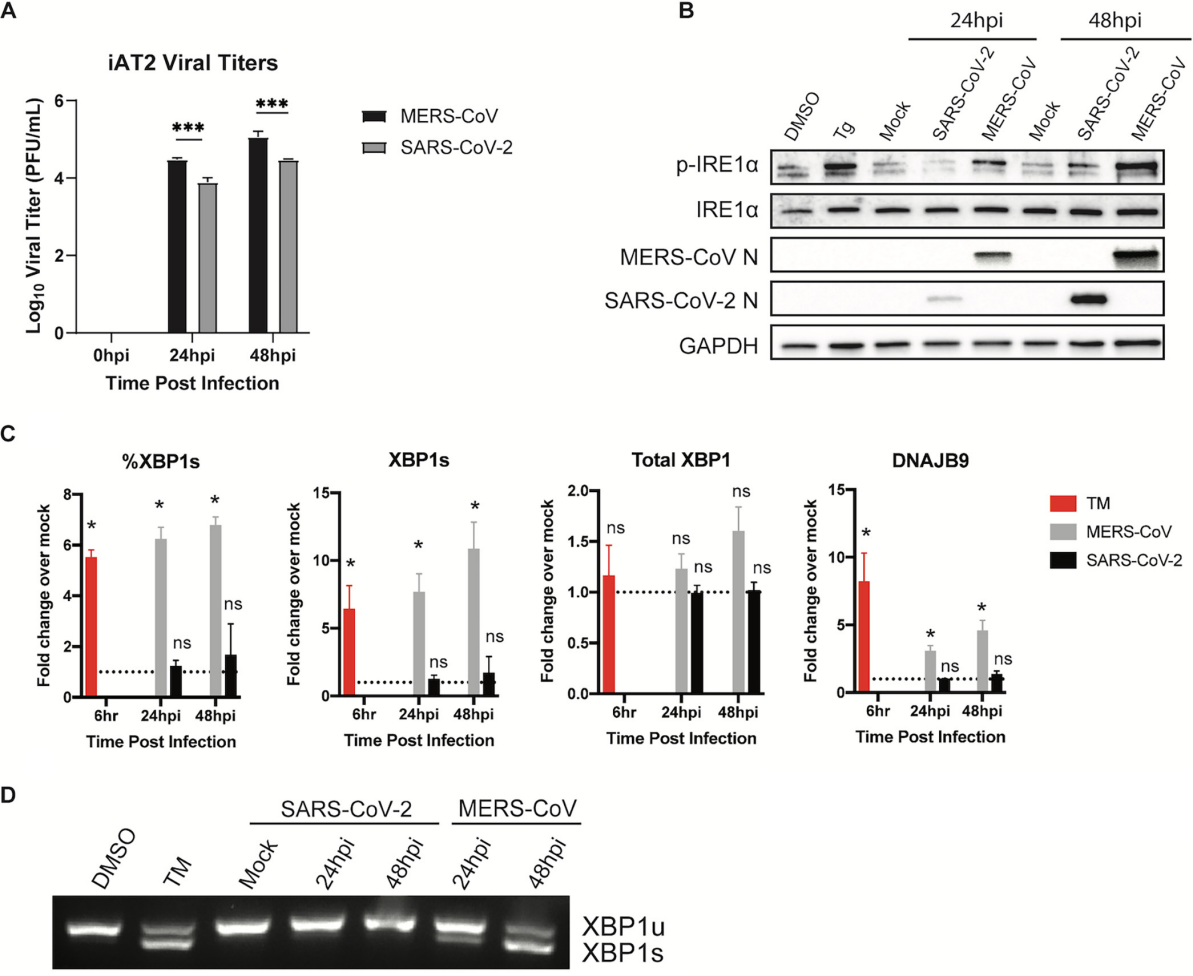
SARS-CoV-2 and MERS-CoV induce IRE1α phosphorylation in iAT2 cells but diverge in induction of XBP1 splicing. iPSC-derived AT2 cells (iAT2 cells) were mock infected or infected (in triplicate) with MERS-CoV or SARS-CoV-2 at an MOI of 5. (A) At the indicated time points, supernatants were collected, and infectious virus was quantified by plaque assay. Values are means ± SD (error bars). Statistical significance was determined by two-way ANOVA (*, *P* < 0.05; ns, not significant). (B) Total protein was harvested at the indicated time points and analyzed by immunoblotting using the indicated antibodies. Thapsigargin treatment for 1 h (Tg; 1 μM) was used as a positive control for IRE1α activation, while DMSO served as a vehicle control. (C) Total RNA was harvested at the indicated time points and relative %XBP1s, XBP1s, and total XBP1 mRNA expression were quantified by RT-qPCR, calculated, and displayed as described above. Values are means ± SD (error bars). Statistical significance (infected compared to mock) was determined using two-tailed, paired Student’s *t* test. Displayed significance is determined by the *P* value; *, *P* < 0.05; **, *P* < 0.01; ***, *P* < 0.001; ****, *P* < 0.0001; ns, not significant. (D) RT-PCR was performed using extracted RNA and primers crossing the XBP1 splicing site. The product was run out on an agarose gel to visualize XBP1 splicing. Tunicamycin treatment (1 μg/mL for 6 h) was used as a positive control for RT-(q)PCR, while DMSO treatment served as a vehicle control. Data shown are from one representative experiment from at least two independent experiments.

Both MERS-CoV and SARS-CoV-2 replicate in these cells and release infectious virus as quantified by plaque assay (Fig. 5A). Notably, MERS-CoV replicated to higher titers than SARS-CoV-2 in these lung-derived cells. This complements our previous findings that SARS-CoV-2 replicates more efficiently than MERS-CoV in upper respiratory-derived primary nasal cells (3) and may suggest that MERS-CoV is better adapted to replicate within the lower respiratory tract while SARS-CoV-2 replicates more efficiently in the upper airway. Despite this difference in replication, both viruses were observed to induce p-IRE1α over the course of infection (Fig. 5B). In agreement with our results in A549 and Calu-3 cells, SARS-CoV-2 failed to induce XBP1 splicing in iAT2 cells, as measured by reverse transcription quantitative PCR (RT-qPCR) (Fig. 5C). In contrast, MERS-CoV induced XBP1 splicing, albeit to a lower extent than in immortalized cell lines. Lastly, we visualized XBP1 splicing using reverse transcriptase PCR (RT-PCR) and agarose gel electrophoresis (Fig. 5D). Again, our data indicate that SARS-CoV-2 fails to induce XBP1 splicing at either 24 or 48 hpi in iAT2 cells, despite inducing p-IRE1α. MERS-CoV, however, induced increasing XBP1 splicing over the course of infection, matching the results in A549 and Calu-3 cells (Fig. 2 and 3). Overall, these results indicate that both SARS-CoV-2 and MERS-CoV induce ER stress as evidenced by IRE1α phosphorylation during infection of primary iAT2 cells, but only MERS-CoV induces the downstream effects of active IRE1α RNase.

### SARS-CoV-2 inhibits XBP1 splicing.

**FIG 6:**
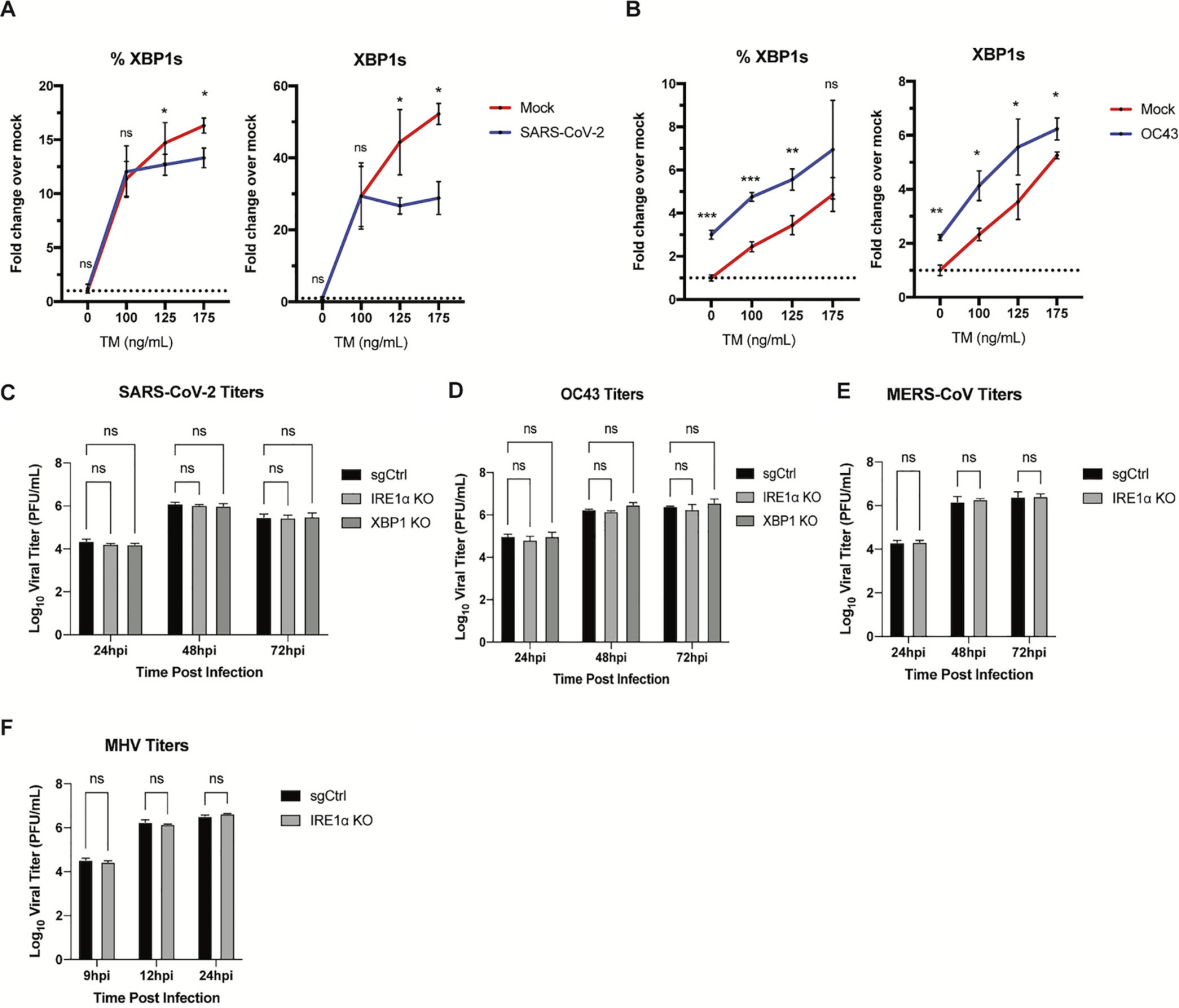
SARS-CoV-2 inhibits IRE1α-mediated XBP1 splicing under ER stress and does not require IRE1α for replication. (A and B) A549-ACE2 cells were mock infected or infected (in triplicate) with SARS-CoV-2 (MOI, 3) (A) or OC43 (MOI, 1) (B) for 24 h prior to treatment with low doses of tunicamycin (100 to 175 ng/mL) for 6 h. Total RNA was harvested and used to quantify the relative %XBP1s and XBP1s expression by RT-qPCR. *C_T_* values were normalized to 18S rRNA and expressed as the fold change over the mock control displayed as 2^−Δ(Δ^*^CT^*^)^. Technical replicates were averaged, and the means for each replicate are displayed as ±SD (error bars). Statistical significance (infected compared to mock) was determined by one-tailed, paired *t* tests (*, *P* < 0.05; **, *P* < 0.01; ***, *P* < 0.001; ns, not significant). (C to F) Infection of CRISPR/Cas9-edited IRE1α KO A549 cells with different coronaviruses. Experiments were performed using sgControl or IRE1α KO or XBP1 KO (where indicated) A549 cells stably expressing viral receptors: A549-ACE2 (OC43 or SARS-CoV-2), A549-DDP4 (MERS-CoV), and A549-MHVR (MHV). Cells were infected (in triplicate) with SARS-CoV-2, MERS-CoV, OC43, or MHV at an MOI of 1. At the indicated times, supernatants were collected, and infectious virus was quantified by plaque assay. Values are means ± SD (error bars). Statistical significance was determined by two-way ANOVA (*, *P* < 0.05; **, *P* < 0.01; ns, not significant). Data shown are from one representative of at least two independent experiments.

We then tested whether SARS-CoV-2 actively inhibits splicing of XBP1 induced by the N-linked glycosylation inhibitor tunicamycin (TM), a common agent used to chemically induce ER stress. To do so, A549-ACE2 cells were either mock infected or infected with SARS-CoV-2 or OC43 for 24 h and then treated with TM for 6 h prior to analysis. Interestingly, while SARS-CoV-2 infection did not completely prevent XBP1 splicing induced by TM, it led to significantly lower XBP1 splicing levels compared with mock infected cells (Fig. 6A). Furthermore, this inhibition is not due to a reduction in phosphorylation of IRE1 (Fig. S3A). In contrast, OC43 increased XBP1 splicing at all tested concentrations of TM (Fig. 6B). This suggests that SARS-CoV-2 actively inhibits activation of the IRE1α RNase.

10.1128/mbio.02415-22.3 FIG S3 Validation of IRE1α and XBP1 KO cell lines using CRISPR/Cas9. (A) A549-ACE2 cells were mock infected or infected (in triplicate) with SARS-CoV-2 (MOI, 3) for 24 h prior to treatment with low doses of tunicamycin (100 to 175 ng/mL) for 6 h. Total protein was harvested and used to quantify phospho-IRE1α and total IRE1α protein levels by Western blotting. Data shown are from one representative experiment of at least three independent experiments. (B to D) A549 cells expressing the indicated viral receptors subjected to CRISPR/Cas9 editing using different guide RNAs targeting IRE1α were immunoblotted for IRE1α protein to assess KO efficiency. (E) CRISPR/Cas-9 gene-edited IRE1α KO A549-ACE2 cells were treated with tunicamycin (500 ng/mL) or DMSO for 6 h. Total RNA was harvested, and %XBP1 was quantified by RT-qPCR. Technical replicates were averaged, and the means for each replicate displayed. Data shown are one representative experiment from at least three independent experiments. (F) CRISPR/Cas9 gene-edited IRE1α KO A549-ACE2 (guide 3) or control A549-ACE2 were treated with tunicamycin (TM; 1 μg/mL) for 8 h. Total RNA was harvested, reverse transcribed, and amplified for XBP1. XBP1 cDNA product was assayed on an agarose gel to visualize splicing. (G) Control or IRE1α KO A549-DDP4 cells were infected with MERS-CoV (MOI, 1). At the indicated time points, total RNA was collected. RT-PCR was performed using primers crossing the XBP1 splicing site. The product was analyzed on an agarose gel to visualize XBP1 splicing. (H) CRISPR/Cas9 gene-edited control or XBP1 KO A549-ACE2 was treated with DMSO or tunicamycin (TM; 1 μg/mL) for 6 h. Lysates were then immunblotted for XBP1s to confirm KO efficiency. Download FIG S3, PDF file, 0.6 MB. Copyright © 2022 Nguyen et al. 2022 Nguyen et al. https://creativecommons.org/licenses/by/4.0/ This content is distributed under the terms of the Creative Commons Attribution 4.0 International license.

### Betacoronaviruses do not require IRE1α for replication.

Given the presumed importance of IRE1α/XBP1s to expand the ER and maintain protein folding during viral replication, and the interesting differences we observed between SARS-CoV-2 and the other betacoronaviruses, we next explored the consequences of its inhibition on the replication of each virus. To determine whether IRE1α activity is required for replication and propagation of MHV, OC43, MERS-CoV, or SARS-CoV-2, we utilized CRISPR/Cas9 gene editing to knock out IRE1α in A549 cell lines expressing receptors for each coronavirus (Fig. S3B to G). Surprisingly, we did not observe any significant differences in the capability of all tested coronaviruses to replicate in cells lacking IRE1α (Fig. 6C to F). These results suggest IRE1α is neither essential nor inhibitory for coronavirus replication in these cells. Since SARS-CoV-2 does not lead to IRE1α-mediated XBP1 splicing, we also tested replication of SARS-CoV-2 and OC43 in XBP1 KO cells (Fig. 6C and D and Fig. S3H). Consistently, there was no detectable effect of XBP1 KO on SARS-CoV-2 or OC43 replication in A549-ACE2 cells. Together, these results demonstrate that none of the coronaviruses tested require the activation IRE1α/XBP1 pathway for optimal replication.

### Loss of IRE1α expression causes robust alterations in gene expression, including reduced interferon signaling, following SARS-CoV-2 infection.

**FIG 7:**
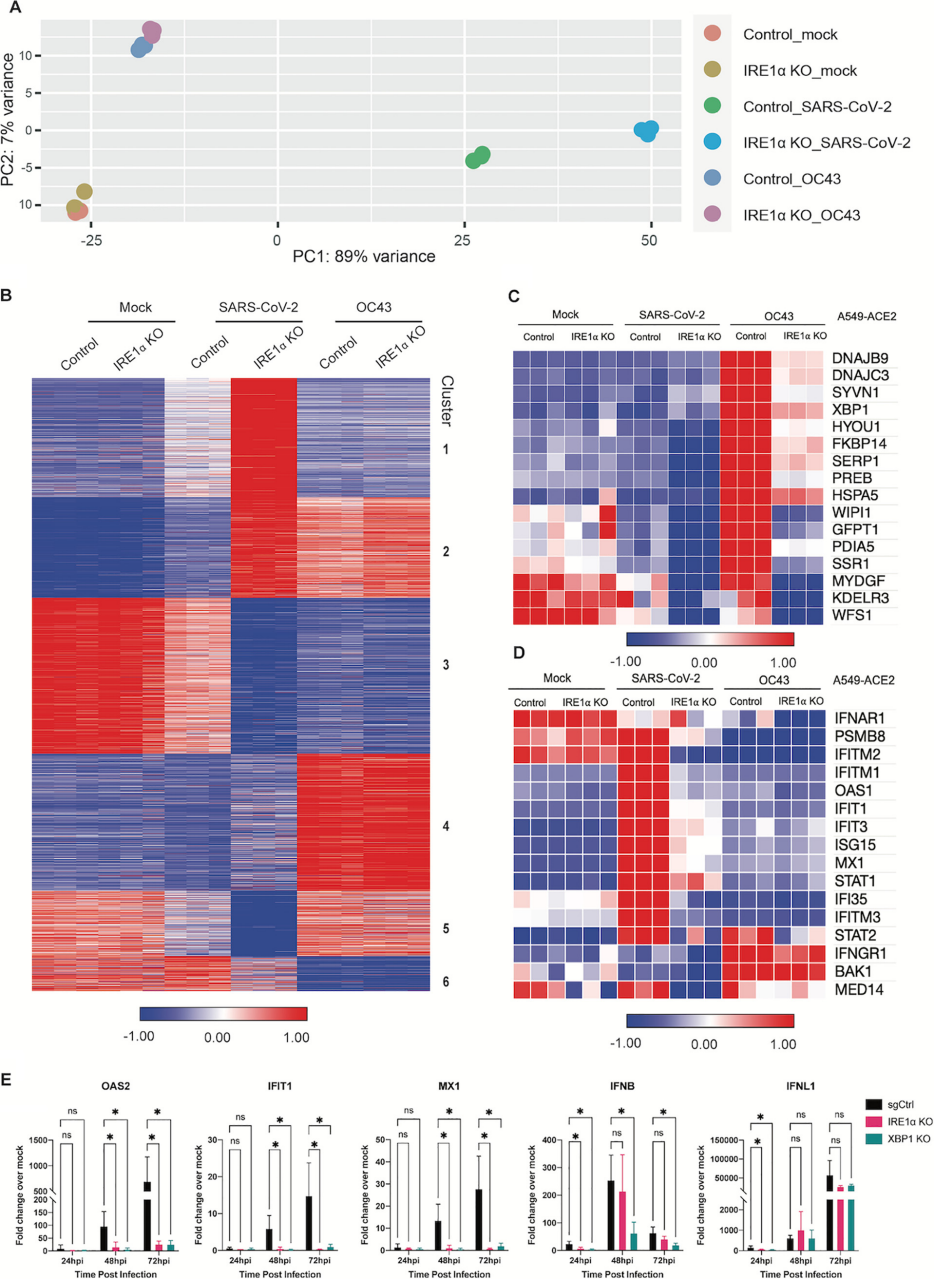
IRE1α promotes the induction of interferon stimulated genes upon SARS-CoV-2 infection. (A to E) A549-ACE2 CRISPR/Cas9-edited IRE1α KO or control cells were mock infected or infected (in triplicate) with SARS-CoV-2 or OC43 (MOI 1) for 48 h. All infections were performed under the same culture conditions at 33°C. Total RNA was harvested, and RNA sequencing was performed as described in Materials and Methods. (A) Principal-component analysis (PCA) of RNA-seq data from samples in triplicate. The first and second principal components (PC1 and PC2) of each sample are plotted. (B) Heatmap of normalized expression levels of the 5,000 most variable genes across all samples were plotted, and K-means clustering was used to divide genes into six clusters based on expression patterns among different treatment conditions. (C and D) Heatmap of normalized expression levels from RNA-seq of ER stress IRE1α-mediated genes (C) or interferon-stimulated genes (D) for all treatment conditions. (E) Total RNA was used to quantify and validate expression of ISGs by RT-qPCR. *C_T_* values were normalized to 18S rRNA and expressed as the fold change over the mock control displayed as 2^−Δ(Δ^*^CT^*^)^. Technical replicates were averaged, and the means for each replicate are displayed as ± SD (error bars). Statistical significance (infected compared to mock) was determined by ordinary one-way ANOVA (*, *P* < 0.05; **, *P* < 0.01; ***, *P* < 0.001; ****, *P* < 0.0001; ns, not significant).

**FIG 8:**
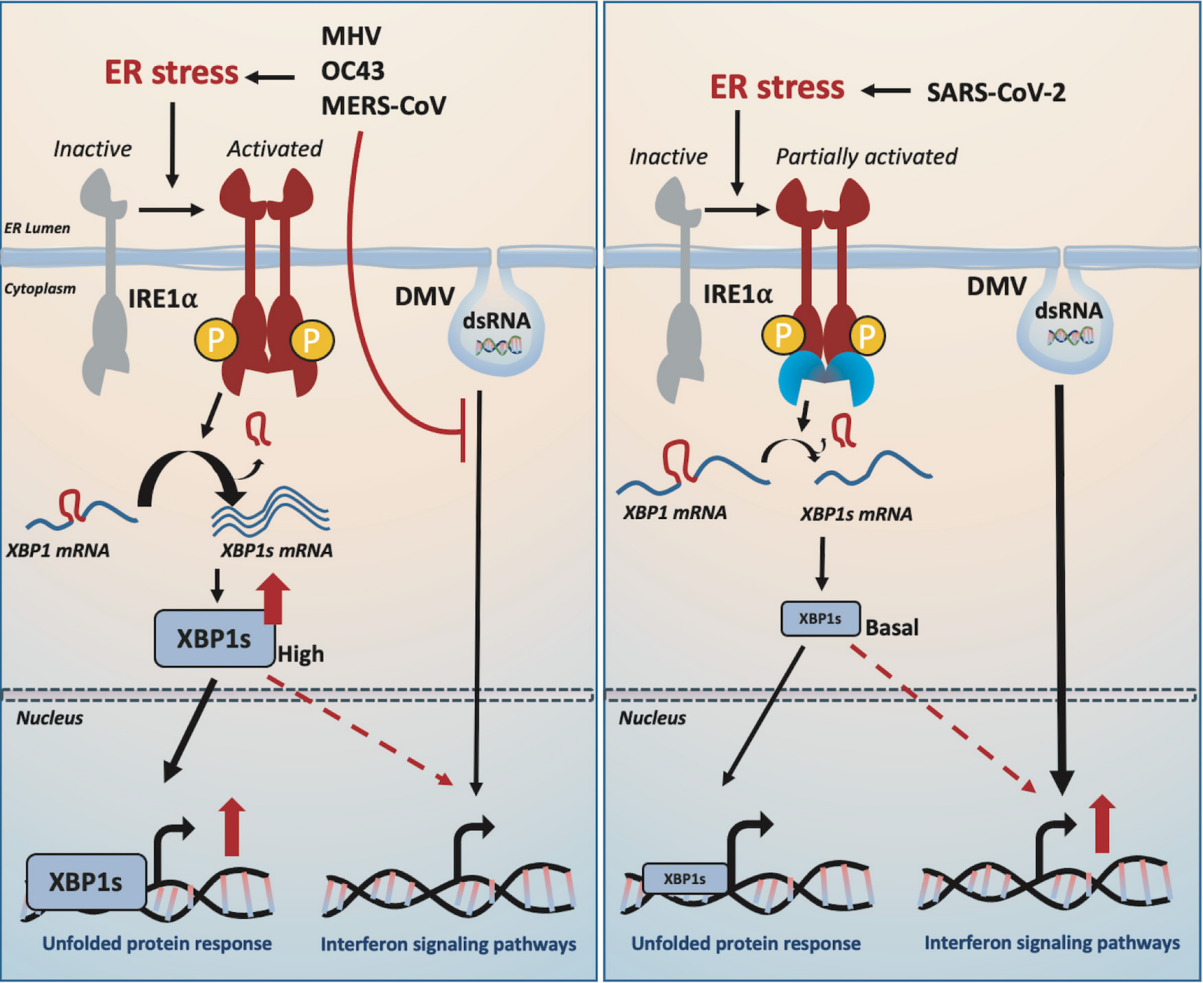
Model of betacoronavirus activation of the IRE1α/XBP1 pathway and downstream effects on interferon signaling. MHV, OC43, and MERS-CoV infection induces ER stress that leads to IRE1α autophosphorylation and downstream IRE1α RNase-mediated XBP1 splicing producing XBP1s. In contrast, SARS-CoV-2 infection only partially activates IRE1α through autophosphorylation but prevents the activation of the RNase activity. XBP1s maintains a low basal level upon SARS-CoV-2 infection. MERS-CoV, OC43, and MHV efficiently antagonize dsRNA induction of IFN signaling. In contrast, SARS-CoV-2 allows dsRNA induction of some IFN signaling, and basal XBP1s potentiates the induction of IFN signaling upon SARS-CoV-2 infection.

To gain insight into the role of IRE1α in regulating betacoronaviruses, we conducted RNA-seq analysis of sg control or IRE1α knockout A549-ACE2 cells infected with either SARS-CoV-2 or OC43 compared to mock-infected cells. Infections of A549-ACE2 cells were carried out at 33°C to enable direct comparison of the two viruses (OC43 replication is significantly more robust at 33°C compared to 37°C [41], while SARS-CoV-2 replicates to a similar extent at both temperatures [Fig. S4A]). Principal-component analysis (PCA) showed a modest change in cellular gene expression upon OC43 infection of wild-type cells relative to SARS-CoV-2, which showed a robust alteration in gene expression (Fig. 7A). In contrast to uninfected or OC43-infected cells, loss of IRE1α significantly impacted host gene expression in SARS-CoV-2-infected A549 cells (Fig. 7A and B). Clustering analysis of RNA-seq data revealed 6 distinct clusters altered upon loss of IRE1α related to key cellular functions, including chromatin organization (cluster 1), mRNA metabolism and processing (cluster 2), and protein translation (cluster 3) (Fig. 7B and Fig. S5A). Detailed analysis of the IRE1α-mediated UPR pathway confirms activation by OC43 infection that is inhibited upon loss of IRE1α (Fig. 7C and Fig. S4B to E). In contrast, minimal change in this pathway was observed in SARS-CoV-2-infected cells, consistent with our previous results in this study. Loss of IRE1α also appears to alter other elements of the UPR in SARS-CoV-2-infected cells, including some genes in the PERK and ATF6 pathways (Fig. S6), which may reflect compensatory effects on the UPR in an attempt to control proteostasis in the absence of IREα (42–44). Strikingly, we observed significantly lower induction of some IFN-stimulated genes (ISGs) during SARS-CoV-2 infection of IRE1α KO cells (Fig. 7D and Fig. S4F and S5B). We have previously reported that SARS-CoV-2 induces type I and type III IFN signaling and ISGs in multiple cell types (3). Interestingly, OC43 infection did not induce notable IFN or ISG responses with or without IRE1α expression, so we were unable to make the same observations with this virus (Fig. 7D). To confirm these results, we performed RT-qPCR on representative IFN genes and ISGs genes that we have previously reported to be upregulated during SARS-CoV-2 infection (3). Consistent with our RNA-seq data, we observed significantly lower induction of ISGs such as OAS2, MX1, and IFIT1 during SARS-CoV-2 infection of cells lacking IRE1α expression at both 37°C (Fig. 7E) and 33°C (Fig. S4F). These data suggest that IRE1α may play a role in augmenting IFN signaling, while not being necessary for ISG induction, in SARS-CoV-2-infected cells. Our data taken together lead us to propose the model shown in Fig. 8.

10.1128/mbio.02415-22.4 FIG S4 IRE1α promotes the induction of IFN-stimulated genes upon SARS-CoV-2 infection. (A) Infection of CRISPR/Cas9-edited IRE1α KO A549-ACE2 cells with OC43 and SARS-CoV-2 (MOI, 1) under the same culture conditions at 33°C. Experiments were performed in triplicate. At the indicated times, supernatants were collected, and infectious virus was quantified by plaque assay. Values are means ± SD (error bars). Statistical significance was determined by two-way ANOVA (ns, not significant). Data shown are from one representative of at least two independent experiments. (B) Quantification of XBP1 splicing by analyzing RNA-seq data (Fig. 7). Reads representing spliced or unspliced XBP1 mRNA were identified based on the presence or absence of the 26-nucleotide intron and quantified. The percentage of XBP1 spliced reads was then plotted. Values are means ± SD (error bars). Statistical significance was determined by ordinary one-way ANOVA. (*, *P* < 0.05; **, *P* < 0.01; ***, *P* < 0.001; ****, *P* < 0.0001; ns, not significant, adjusted after Tukey’s multiple comparisons test). (C and D) Gene set enrichment analysis (GSEA) of IRE1α-mediated unfolded protein response genes with normalized enrichment score (NES) and *P* values compared between IRE1α KO and control cells infected with OC43 (C) or SARS-CoV-2 (D). (E) GSEA of genes that belong to GO term response to type I interferon (left) or response to interferon alpha (right) compared between IRE1α KO and control cells infected with SARS-CoV-2. (F) Infection of IRE1α KO or control A549-ACE2 with SARS-CoV-2 (MOI, 1) at 33°C. At the indicated times post infection, total RNA was collected and gene expression was quantified by RT-qPCR. *C_T_* values were normalized to 18S rRNA and expressed as the fold change over the mock control displayed as 2^−Δ(Δ^*^CT^*^)^. Technical replicates were averaged, and the means for each replicate are displayed as ±SD (error bars). Statistical significance (infected compared to mock) was determined by ordinary one-way ANOVA (*, *P* < 0.05). Download FIG S4, PDF file, 1.2 MB. Copyright © 2022 Nguyen et al. 2022 Nguyen et al. https://creativecommons.org/licenses/by/4.0/ This content is distributed under the terms of the Creative Commons Attribution 4.0 International license.

10.1128/mbio.02415-22.5 FIG S5 Metascape analysis of SARS-CoV-2 and OC43 infection RNA-seq data. (A) Metascape analyses of genes from six clusters (Fig. 7B). GO terms and KEGG pathways (hsa) are shown with –log 10 *P* values. (B) Ingenuity-generated interferon signaling pathway analysis comparing IRE1α KO to control cells upon SARS-CoV-2 infection from RNA-seq results (Fig. 7). Upregulated genes (red), downregulated genes (green), or no significant differential expression genes (gray) are shown with color intensity corresponding to log 2 fold-change values from RNA-seq data. Download FIG S5, PDF file, 1.3 MB. Copyright © 2022 Nguyen et al. 2022 Nguyen et al. https://creativecommons.org/licenses/by/4.0/ This content is distributed under the terms of the Creative Commons Attribution 4.0 International license.

10.1128/mbio.02415-22.6 FIG S6 Transcriptomic changes in the host canonical pathway of unfolded protein response upon SARS-CoV-2 and OC43 infection. (A to C) Heatmap of normalized expression levels from RNA-seq (Fig. 7) of genes from the canonical pathway of the UPR (A), PERK branch of UPR (B), or ATF6 branch of UPR (C). Download FIG S6, PDF file, 0.2 MB. Copyright © 2022 Nguyen et al. 2022 Nguyen et al. https://creativecommons.org/licenses/by/4.0/ This content is distributed under the terms of the Creative Commons Attribution 4.0 International license.

## DISCUSSION

Human respiratory betacoronaviruses initiate infection in the upper respiratory tract and have the potential to cause life-threatening pneumonia as a result of infection and inflammation of the lower respiratory tract. The host response to severe infection with coronaviruses is associated with marked dysfunction in the distal lung (alveolar) epithelium, which includes disruption of barrier function, dysregulated immune responses, transcriptomic reprogramming to a transitional cell state, and senescence (45, 46).

To better understand the host epithelial response to coronavirus infection, we systematically compared the activation of the IRE1α/XBP1 pathway of the UPR during infection with betacoronaviruses in lung-derived A549 and Calu-3 cells lines and iPSC-derived AT2 cells. We employed three human viruses, each from a different betacoronavirus subgenus, OC43 (embeco), SARS-CoV-2 (sarbeco) and MERS-CoV (merbeco), and included the murine coronavirus MHV, a model embecovirus. We found a striking difference between the host response to SARS-CoV-2 and the other three viruses. OC43, MHV, and MERS-CoV all activated the canonical IRE1α/XBP1 pathway in both A549 and Calu-3 cell lines as evidenced by phosphorylation of IRE1α (Fig. 2), XBP1 mRNA splicing (Fig. 3 and 4) and induction of DNAJB9 (Fig. 3), a transcriptional target of XBP1s. Additionally, MERS-CoV was observed to induce IRE1α/XBP1 activation in iAT2 cells (Fig. 5). In contrast, while SARS-CoV-2 also promoted autophosphorylation of IRE1α, there was no evidence of XBP1s, indicating that the pathway was only partially activated and suggesting that the IRE1α kinase was active while the XBP1 splicing RNase activity was not. The differential splicing of XBP1 mRNA during SARS-CoV-2 and MERS-CoV infection was also observed in iPSC-derived AT2 cells, confirming the results in a more physiologically relevant system (Fig. 5). The difference among these viruses is surprising, as all of them encode highly conserved replicase and structural proteins that promote ER membrane rearrangements and challenge the ER folding capacity, respectively (32). We had originally hypothesized that these conserved genes would induce similar stress on the ER and lead to UPR activation. Instead, our data suggest that that SARS-CoV-2 actively prevents XBP1 splicing (Fig. 6A and B). Consistent with this idea, a recombinant SARS-CoV lacking the E protein (rSARS-CoV-ΔE) was reported to induce more XBP1 splicing as well as induction of UPR genes compared to parental wild-type virus (47).

To investigate the importance of IRE1α for coronavirus replication, we evaluated replication of each of the betacoronaviruses in IRE1α KO A549 cells compared to parental wild-type cells. In contrast to influenza (48), all of the betacoronaviruses examined were able to replicate efficiently in the absence of IRE1α signaling, consistent with a previous report of the gammacoronavirus IBV (31). While we did observe a decrease in OC43 and SARS-CoV-2 nucleocapsid expression following KIRA8 treatment (Fig. 2A and F), the similar levels of replication of all the viruses in IRE1α KO cells and parental cells (Fig. 6C to F) suggest that this is due to off-target effects of KIRA8 rather than IRE1α inhibition limiting virus replication. This raises interesting possibilities for the role of IRE1α during coronavirus infection. As previously stated, IRE1α can produce both cytoprotective (through XBP1s) and destructive responses (via RIDD and JNK/p38 signaling) depending on the extent of the encountered stress. It seems likely that coronavirus infection would induce extensive and prolonged ER stress, which may push IRE1α beyond the initial pro-recovery responses and toward a pro-apoptotic response. Indeed, our data reveal that, at least with MERS-CoV and SARS-CoV-2 infection, IRE1α phosphorylation is readily detectable by 24 hpi and remains steady throughout the course of infection (Fig. S1A and B). Additionally, unlike what has been observed with chemically induced ER stress (36, 49), IRE1α phosphorylation does not appear to attenuate at any point during coronavirus infection, again suggesting a hyperactive and destructive outcome. As stated above, destruction of cells, in particular, AT2 cells in the lung, may contribute to pathogenesis during coronavirus infection. However, SARS-CoV-2 appears to limit the downstream consequences of IRE1α activation, most notably, XBP1 splicing via its RNase activity, and thus may be protected from this destructive phenotype. MERS-CoV may induce apoptosis redundantly in the UPR, as it has been reported that MERS-CoV induces and benefits from apoptosis mediated by the PERK arm of the UPR (27, 50).

To further probe the impact of IRE1α signaling on host gene expression following coronavirus infection, we performed RNA-seq analysis of sg control or IRE1α knockout A549-ACE2 cells infected with either SARS-CoV-2 or OC43. IRE1α deletion significantly reduced the expression of genes downstream of XBP1s during OC43 infection, as expected, with otherwise only modest changes in overall gene expression. In contrast, genetic ablation of IRE1α significantly impacted host gene expression in SARS-CoV-2-infected A549 cells. The two most dramatic effects that appear to be specific to SARS-CoV-2 relate to chromatin organization and protein folding and transport. Effects on mRNA metabolism and processing are also observed for SARS-CoV-2 and, more modestly, for OC43. Finally, protein translation is downregulated in both OC43 and SARS-CoV-2-infected cells but, in the latter case, occurs primarily upon loss of IRE1α. Taken together, these results suggest that IRE1α plays a key role in mediating changes in host cell gene transcription and protein production caused by SARS-CoV-2.

We found here that deletion of IRE1α blunted the induction of some but not all ISGs by SARS-CoV-2 infection. In contrast, OC43 was not observed to induce significant levels of IFN or ISG mRNAs in either WT or IRE1α KO cells. The mechanism by which loss of IRE1α activity during SARS-CoV-2 infection dampens the induction of interferon signaling remains to be determined. It has been reported that the UPR can precede and prime innate immune signaling in flavivirus-infected cells (51). XBP1s has been found upstream of IFNα and IFNβ transcription and may work through binding upstream *cis*-acting enhancer elements (52, 53). Moreover, XBP1s can directly bind and transcriptionally activate interleukin-6 (IL-6), tumor necrosis factor α (TNF-α), and other inflammatory cytokines (54). It is possible that a low level of background XBP1 splicing may occur during SARS-CoV-2 infection, which could contribute to these responses. Independent of its RNase activity, the autophosphorylated cytoplasmic domain of IRE1α can oligomerize and serve as a scaffold that recruits TRAF2, JNK, ASK, Nck, and other molecules that can lead to varied signaling outputs (55, 56). Therefore, the ability of SARS-CoV-2 to prevent full IRE1α activation might dampen inflammatory signaling and prevent detection and elimination by the immune system in an intact organism. However, it is important to note that the diminution of ISG expression in the absence of IRE1α is variable among ISGs, and SARS-CoV-2 still induces IFN and IFN signaling to a greater extent than OC43 in IRE1α KO cells. We speculate that SARS-CoV-2 has adapted to tolerate a low level of IFN signaling as well as protein kinase R (PKR) and oligoadenylate RNase L (OAS/RNase L) activation, and the reduced ISG expression in the absence of IRE1α does not have enough of an effect to promote increased replication. This is consistent with our finding that knockout of mitochondrial antiviral signaling protein (MAVS) from A549 cells, resulting in minimal IFN expression and ISG signaling, does not promote increased SARS-CoV-2 replication (3). Thus, the significance of IRE1α-dependent IFN signaling is not clear and will be a subject of future investigation.

Overall, despite the lack of apparent virus replication defects with IRE1α deficiency, further characterization of the repertoire of betacoronavirus-induced IRE1α signaling is warranted, including contributions to cytokine production, apoptosis, and proinflammatory responses. While we initially investigated this pathway from the perspective of the impact on virus replication, future studies should examine effects of IRE1α activation on the host, including inflammation and cell death through the JNK and p38 mitogen-activated protein kinase (MAPK) signaling scaffolded by IRE1α (22) and/or RIDD, as a consequence of prolonged IRE1α activation (17, 57). These responses could be particularly important in AT2 cells, which must rely on the UPR to maintain proteostasis in the face of the challenge from the biosynthesis and secretion of surfactant proteins (58). Dysregulation of these responses by coronavirus infection could promote AT2 cell reprogramming, epithelial apoptosis, alteration of surfactant components in alveoli, and the rampant inflammation associated with severe coronavirus infection (59–61). Finally, the UPR response is complex and made up of the PERK and ATF6 pathways in addition to IRE1α, and signals from all three of these pathways almost certainly integrate into the final outcome of an infected cell. Indeed, changes in the PERK and ATF6 pathways may compensate for the IRE1α deficiency in the KO cells and explain the absence of an effect on replication of any of the betacoronaviruses under study.

We recently reported that SARS-CoV-2 and MERS-CoV also diverge in their activation and antagonism of the dsRNA-induced host cell innate immune responses, another early innate response to viruses (3). While MERS-CoV actively antagonizes type I and type III interferon production and signaling, the oligoadenylate RNase L (OAS/RNase L) system and the PKR pathway, SARS-CoV-2 activates OAS/RNase L and PKR and induces a low level of IFN and ISG expression (3, 4) in A549 and Calu-3 respiratory tract-derived cells. Here, we observed that OC43 infection did not lead to the induction of IFN or ISGs (Fig. 7D), and we have shown previously that OC43-encoded accessory protein NS2 antagonizes activation of the OAS/RNase L pathway (62). Activation of these pathways during MERS-CoV mutant infection significantly reduces virus replication (63), while SARS-CoV-2 can tolerate the innate responses activated during infection (3).

Considering the differences we have observed between betacoronaviruses with innate immune responses and now IRE1α activation and signaling, it is striking that MERS-CoV and SARS-CoV-2 are reciprocal in what they activate and antagonize. To optimize replication, coronaviruses must likely strike a balance in the cellular responses they antagonize, tolerate, or benefit from. Supporting this, our data suggest that IRE1α influences ISG induction during infection. It is intriguing to consider if MERS-CoV tolerates this by antagonizing IFN and ISG induction, while SARS-CoV-2 instead limits IRE1α activity. Future studies should examine the synergy between innate immune responses and the UPR during coronavirus infection and how perturbations on one side may change viral replicative capacity, tropism, and spread. Understanding how signals from each one of these pathways are integrated into viral replication and cell fate decisions during coronavirus infection may illuminate new therapeutic strategies for combating emerging betacoronaviruses.

## MATERIALS AND METHODS

### Cell lines.

Human A549 cells (ATCC CCL-185) and its derivatives were cultured in RPMI 1640 (Gibco catalog no. 11875) supplemented with 10% fetal bovine serum (FBS), 100 U/mL penicillin, and 100 μg/mL streptomycin (Gibco catalog no. 15140). African green monkey kidney Vero cells (E6) (ATCC CRL-1586) and VeroCCL81 cells (ATCC CCL-81) were cultured in Dulbecco’s modified Eagle’s medium (DMEM; Gibco catalog no. 11965) supplemented with 10% FBS, 100 U/mL of penicillin, 100 μg/mL streptomycin, 50 μg/mL gentamicin (Gibco catalog no. 15750), 1 mM sodium pyruvate (Gibco catalog no. 11360), and 10 mM HEPES (Gibco catalog no. 15630). Human HEK 293T cells (ATCC CRL-3216) were cultured in DMEM supplemented with 10% FBS. Human Calu-3 cells (ATCC HTB-55) were cultured in DMEM supplemented with 20% FBS without antibiotics. Mouse L2 cells (64) were grown in DMEM supplemented with 10% FBS, 100 U/mL penicillin, 100 μg/mL streptomycin, 10 nM HEPES, 2 mM l-glutamine (Gibco catalog no. 25030081), and 2.5 μg/mL amphotericin B (Gibco catalog no. 15290).

A549-DPP4 (4), A549-ACE2 (3), and A549-MHVR (4) cells were generated as described previously. A549-ACE2 cells, used in Fig. 3I and J, Fig. 4, Fig. 6, and Fig. S3 were a kind gift of Benjamin TenOever, Mt. Sinai Icahn School of Medicine. CRISPR-Cas9 knockout cell lines were generated using lentiviruses. Lentivirus stocks were generated by using lentiCRISPR v2 (Addgene) with single guide RNA (sgRNA) targeting IRE1α sequences (version 1 [V1]: CGGTCACTCACCCCGAGGCC, V2: TTCAGGAAGCGTCACTGTGC, V3: CGGTCACTCACCCCGAGGCC) or XBP1 sequence (TCGAGCCTTCTTTCGATCTC). The infected A549-ACE2 cells were polyclonally selected and maintained by culture in medium supplemented with 4 μg/mL puromycin for 1 week.

iPSC (SPC2 iPSC line, clone SPC2-ST-B2, Boston University)-derived alveolar epithelial type 2 cells (iAT2) were grown and infected as previously described (3). In brief, cells were differentiated and maintained as alveolospheres embedded in 3D Matrigel in CK+DCI medium, as previously described (65). For generation of 2D alveolar cells for viral infection, alveolospheres were dispersed into single cells and then plated on precoated 1/30 Matrigel plates at a cell density of 125,000 cells/cm^2^ using CK+DCI medium with ROCK inhibitor for the first 48 h, and then the medium was changed to CK+DCI medium at day 3 and either mock infected or infected with MERS-CoV or SARS-CoV-2 at an MOI of 5.

### Viruses.

SARS-CoV-2 (USA-WA1/2020) was obtained from BEI Resources, NIAID, NIH or provided by Natalia Thornburg, World Reference Center for Emerging Viruses and Arboviruses (Galveston, Texas) and propagated in VeroE6-TMPRSS2 cells. The genomic RNA was sequenced and found to be identical to that of GenBank version no. MN985325.1. Recombinant MERS-CoV was described previously (1) and propagated in VeroCCL81 cells. SARS-CoV-2 and MERS-CoV infections were performed at the University of Pennsylvania or at the Howard Taylor Ricketts Laboratory (HTRL) at Argonne National Laboratory (Lemont, IL) in biosafety level 3 (BSL-3) laboratories under BSL-3 conditions, using appropriate and approved personal protective equipment and protocols. OC43 was obtained from ATCC (VR-1558) and grown and titrated on VeroE6 cells at 33°C or on A549-mRuby cells as previously described (66). MHV-A59 (5, 67) was propagated on A549-MHVR cells or on murine 17CL-1 cells.

### Viral growth kinetics and titration.

SARS-CoV-2 and MERS-CoV infections and plaque assays were performed as previously described (1, 5). In brief, A549 cells were seeded at 3 × 10^5^ cells per well in a 12-well plate for infections. Calu-3 cells were seeded similarly onto rat tail collagen type I-coated plates (Corning no. 356500). Cells were washed once with phosphate-buffered saline (PBS) before being infected with virus diluted in serum-free medium—RPMI for A549 cells or DMEM for Calu-3 cells. Virus was absorbed for 1 h (A549 cells) or 2 h (Calu-3 cells) at 37°C before the cells were washed 3 times with PBS and the medium was replaced with 2% FBS RPMI (A549 cells) or 4% FBS DMEM (Calu-3 cells). At the indicated time points, 200 μL of medium was collected to quantify released virus by plaque assay and stored at −80°C. Infections for MHV growth curves were performed similarly under BSL-2 conditions. For OC43 infections, similar infection conditions and media were used; however, virus was absorbed, and the infections were incubated at 33°C rather than 37°C.

Plaque assays were performed using VeroE6 cells for SARS-CoV-2 and OC43, VeroCCL81 cells for MERS-CoV, and L2 cells for MHV. SARS-CoV-2 and MERS-CoV plaque assays were performed in 12-well plates at 37°C. OC43 and MHV plaque assays were performed in 6-well plates at 33°C and 37°C, respectively. In all cases, virus was absorbed onto cells for 1 h at the indicated temperatures before overlay was added. For SARS-CoV-2, MERS-CoV, and OC43 plaque assays, a liquid overlay was used (DMEM with 2% FBS, 1× sodium pyruvate, and 0.1% agarose). A solid overlay was used for MHV plaque assays (DMEM plus 2% FBS, 1× HEPES, 1× glutamine, 1× Fungizone, and 0.7% agarose). Cell monolayers were fixed with 4% paraformaldehyde and stained with 1% crystal violet after the following incubation times: SARS-CoV-2 and MERS-CoV, 3 days; OC43, 5 days; MHV, 2 days. All plaque assays were performed in biological triplicate and technical duplicate.

### Pharmacologic agents.

KIRA8 was purchased at >98% purity from Chemveda Life Sciences India Pvt. Ltd. For use in tissue culture, KIRA8 stock solution was prepared by dissolving in dimethyl sulfoxide (DMSO). Tunicamycin (catalog no. T7765) and Tg (catalog no. T9033) were purchased at >98% purity from Sigma. For use in tissue culture, tunicamycin and TG stock solutions were prepared by dissolving in DMSO.

### Immunoblotting.

Cells were washed once with ice-cold PBS, and lysates were harvested at the indicated times post infection with lysis buffer (1% NP-40, 2 mM EDTA, 10% glycerol, 150 mM NaCl, 50 mM Tris HCl, pH 8.0) supplemented with protease inhibitors (Roche complete mini-EDTA-free protease inhibitor) and phosphatase inhibitors (Roche PhosStop easy pack). After 5 min, lysates were incubated on ice for 20 min and centrifuged for 20 min at 4°C, and supernatants were mixed 3:1 with 4× Laemmli sample buffer (Bio-Rad 1610747). Samples were heated at 95°C for 5 min and then separated on SDS-PAGE and transferred to polyvinylidene difluoride (PVDF) membranes. Blots were blocked with 5% nonfat milk or 5% bovine serum albumin (BSA) and probed with antibodies (Table 1) diluted in the same blocking buffer. Primary antibodies were incubated overnight at 4°C or for 1 h at room temperature. All secondary antibody incubation steps were done for 1 h at room temperature. Blots were visualized using Thermo Scientific SuperSignal chemiluminescent substrates (catalog no. 34095 or 34080). The antibodies are listed in Table 1.

**TABLE 1:** Antibodies

Primary antibody	Antibody species	Blocking buffer^*a*^	Dilution	Catalog no.
Phospho-IRE1α	Rabbit	5% BSA/TBST	1:1,000	Abcam EPR5253 Invitrogen PA585738
IRE1α (14C10)	Rabbit	5% Milk/TBST	1:1,000	Cell Signaling Technology 3294S
XBP1s	Mouse	5% Milk/TBST	1:1,000	BioLegend 9D11A43
GAPDH (14C10)	Rabbit	5% Milk/TBST	1:2,000	Cell Signaling Technology 2118S
SARS-CoV-2 N	Rabbit	5% Milk/TBST	1:2,000	Gentex GTX135357
MERS-CoV N	Mouse	5% Milk/TBST	1:2,000	Sino Biological40068-MM10
OC43 N	Rabbit	5% Milk/TBST	1:2,000	Sino Biological 40643-T62

a TBST, Tris-buffered saline with Tween 20.

### RNA sequencing.

A549 cells expressing the MERS-CoV receptor DPP4 (4) were cultured in 10% FBS RPMI medium. At 70% cell confluence, cells were washed once with PBS before being mock infected or infected with MERS-CoV (EMC/2012) at and MOI of 1. Virus was absorbed for 1 h at 37°C in serum-free RPMI medium. After 1 h, virus was removed, cells were washed three times with PBS, and 2% FBS RPMI was added. The cells were incubated for another 24 h or 36 h and then washed once with PBS and lysed using RLT Plus lysis buffer before genomic DNA removal and total RNA extraction using the Qiagen RNeasy Plus minikit (Qiagen 74134). Three independent biological replicates were performed per experimental condition. RNA sample quality check, library construction, and sequencing were performed with GeneWiz following standard protocols. All samples were sequenced using an Illumina HiSeq sequencer to generate paired-end 150-bp reads. Read quality was assessed using FastQC v0.11.2 as described in reference 68. Raw sequencing reads from each sample were quality and adapter trimmed using BBDuk 38.73 as described in reference 69. The reads were mapped to the human genome (hg38 with Ensembl v98 annotation) using RNA STAR v2.7.1a (70). The resulting BAM files were counted with featureCounts v1.6.4 to count the number of reads for each gene (71). Differential expression between mock, 24 hpi, and 36 hpi experimental conditions were analyzed using the raw gene counts files by DESeq2 v1.22.1 (72). A PCA plot of RNA-seq samples and a normalized gene expression matrix were also generated with DESeq2.

For SARS-CoV-2 and OC43 infections, ACE2-A549 sg control or IRE1 KO cells were cultured in 10% FBS RPMI to 70% confluence. Cells were washed once with PBS before being mock infected or infected with each virus at an MOI of 1 for 1 h in serum-free RPMI at 33°C. Cells were then washed three times with PBS before 2% FBS RPMI was added. At 48 hpi, cells were lysed with RLT Plus lysis buffer before genomic DNA removal and total RNA extraction using the Qiagen RNeasy Plus minikit (Qiagen 74134). Three independent biological replicates were performed per experimental condition. RNA sample quality check, library construction, and sequencing were performed by the University of Chicago Genomics Facility following standard protocols. All samples were sequenced in two runs using a NovaSeq 6000 sequencer to generate paired-end 100-bp reads. For each sample, the reads from two flow cells were combined before downstream processing. Quality and adapter trimming were performed on the raw sequencing reads using TrimGalore v0.6.3 (https://github.com/FelixKrueger/TrimGalore). The reads were mapped to the human genome (UCSC hg19 with GENCODE annotation), and the downstream analyses were performed using the same methods as described above.

### Host pathway activity analysis of viruses.

RNA-seq data from Gene Expression Omnibus (GEO) no. GSE147507 (37), GSE168797 (38), and GSE144882 (35) and the data presented herein were used to compare the effects of different viruses on host ER stress response. Specifically, Ingenuity Pathway Analysis (IPA) (https://digitalinsights.qiagen.com/products-overview/discovery-insights-portfolio/analysis-and-visualization/qiagen-ipa/) was used to predict activities of related canonical pathways based on host gene expression changes following viral infection. Activation Z-scores for every virus and canonical pathway combination were plotted as a heatmap using Morpheus (https://software.broadinstitute.org/morpheus). IPA used the following *q* value cutoffs for each data set to perform the canonical pathway cross-comparison: Calu-3 SARS-CoV-2 MOI 2 24 h *q* < 0.05, NHBE SARS-CoV-2 MOI 2 24 h *q* < 0.1, A549-ACE2 SARS-CoV-2 MOI 0.2 24 h *q* < 0.1, A549-ACE2 SARS-CoV-2 MOI 2 24 h *q* < 0.05, A549-ACE2 SARS-CoV-2 MOI 3 24 h *q* < 0.01, A549-ACE2 SARS-CoV-2 MOI 1 48 h 33°C *q* < 0.05, A549-ACE2 OC43 MOI 1 48 h 33°C *q* < 0.001, A549-DPP4 MERS-CoV MOI 1 24 h *q* < 0.1, A549-DPP4 MERS-CoV MOI 1 36 h *q* < 0.01, BMDM MHV-A59 MOI 1 12 h *q* < 0.1 and over 1-fold up- or downregulated. These cutoffs were implemented due to the limitations set by the IPA software. IPA was also used to overlay gene expression data (log_2_ fold change) onto the interferon signaling pathway map (Fig. S5B).

### Gene expression heatmaps.

Expression levels for genes involved in various pathways from RNA-seq data were drawn using Morpheus. For each gene, the normalized expression values of all samples were transformed by subtracting the mean and dividing by the standard deviation. The transformed gene expression values were used to generate the heatmap. For the clustering analysis of RNA-seq experiments for OC43- and SARS-CoV-2-infected A549-ACE2 cells with or without IRE1α, the top 5,000 most variable genes were selected. The normalized gene expression data were analyzed using Morpheus. K-means clustering with 6 clusters was applied to the gene expression data.

### Gene set enrichment analyses.

To identify themes across the 6 clusters, functional gene set enrichment analyses for the genes in each cluster were performed using Metascape (73). The following categories were selected for the enrichment analyses: GO molecular functions, GO biological processes, and KEGG pathway. Metascape analysis was performed with a minimum *P* value significance threshold of 0.05, a minimum overlap of 10 genes, and a minimum enrichment score of 5. Notable pathways enriched by Metascape from each cluster were summarized in a heatmap using Morpheus. GSEA v4.1.0 (74) was used to perform specific gene set enrichment analyses on Gene Ontology terms: IRE1-mediated unfolded protein response (75, 76), response to type I interferon (77), and response to interferon alpha (78) using the normalized expression data from the RNA-seq experiment for OC43- and SARS-CoV-2-infected A549-ACE2 cells with or without IRE1α.

### Statistical analysis.

All statistical analyses and plotting of data were performed using GraphPad Prism software. RT-qPCR data were analyzed by Student’s *t* test. Plaque assay data were analyzed by two-way analysis of variance (ANOVA) with multiple-comparison correction. Displayed significance is determined by the *P* value; *, *P* < 0.05; **, *P* < 0.01; ***, *P* < 0.001; ****, *P* < 0.0001; ns, not significant.

### Quantification of XBP1 alternative splicing using RNA-seq data.

BAM files produced using RNA STAR were analyzed in Integrative Genomics Viewer v2.9.4 to count the number of XBP1 reads containing the alternative splicing (79). The total number of XBP1 reads was counted with featureCounts. The percentage of XBP1 alternative splicing for each sample was determined by dividing the number of alternatively spliced reads by the number of total XBP1 reads (spliced plus unspliced).

### Quantitative PCR (RT-qPCR).

Cells were lysed with RLT Plus buffer, and total RNA was extracted using the RNeasy Plus minikit (Qiagen). RNA was reverse transcribed into cDNA with a high-capacity cDNA reverse transcriptase kit (Applied Biosystems 4387406). cDNA samples were diluted in molecular biology-grade water and amplified using specific RT-qPCR primers (see Table 2). RT-qPCR experiments were performed on a Roche LightCycler 96 instrument. SYBR green supermix was from Bio-Rad. Host gene expression displayed as the fold change over mock-infected samples was generated by first normalizing cycle threshold (*C_T_*) values to 18S rRNA to generate Δ*C_T_* values (Δ*C_T_* = *C_T_* gene of interest − *C_T_* 18S rRNA). Next, Δ (Δ*C_T_*) values were determined by subtracting the mock-infected Δ*C_T_* values from the virus-infected samples. Technical triplicates were averaged and means displayed using the equation 2^–Δ(Δ^*^CT^*^)^. Primer sequences are listed in Table 2.

**TABLE 2:** Primer sequences

Target sequence	Forward primer (5′ to 3′)	Reverse primer (5′ to 3′)
XBP1s	GCTGAGTCCGCAGCAGGT	CTGGGTCCAAGTTGTCCAGAAT
XBP1 total	TGAAAACAGAGTAGCAGCTCAGA	CCCAAGCGCTGTCTTAACTC
RPL13A	CTCAAGGTGTTTGACGGCATCC	TACTTCCAGCCAACCTCGTGAG
18S rRNA	TTCGATGGTAGTCGCTGTGC	CTGCTGCCTTCCTTGAATGTGGTA
SARS-CoV-2 genome (nsp12/RdRp)	GGTAACTGGTATGATTTCG	CTGGTCAAGGTTAATATAGG
MERS-CoV genome (nsp7)	GCACATCTGTGGTTCTCCTCTCT	AAGCCCAGGCCCTACTATTAGC
DNAJB9	AGTCGGAGGGTGCAGGATATT	TTGATTTGGCGCTCTGATGC

### XBP1 splicing assay by RT-qPCR.

RT-qPCR was used to quantify the relative expression of the spliced version of XBP1 (XBP1s) by using specific pairs of primers for human alternatively spliced XBP1 and total XBP1 (primer sequences are described above) as previously described (80). The relative percentage of alternative splicing of XBP1 (%XBP1s) was indicated by calculating the ratio of signals between XBP1s and total XBP1.

### Data availability.

Raw and processed RNA-seq data for MERS-CoV, OC43, and SARS-CoV-2 were deposited in the Gene Expression Omnibus database (GSE193169).
