# Supplementary figures and images for: SARS-CoV-2 Diverges from Other Betacoronaviruses in Only Partially Activating the IRE1α/XBP1 Endoplasmic Reticulum Stress Pathway in Human Lung-Derived Cells
Source: mBio. 2022 Sep 20;13(5):e02415-22. doi: 10.1128/mbio.02415-22 (PMC9600248; doi:10.1128/mbio.02415-22)

**A**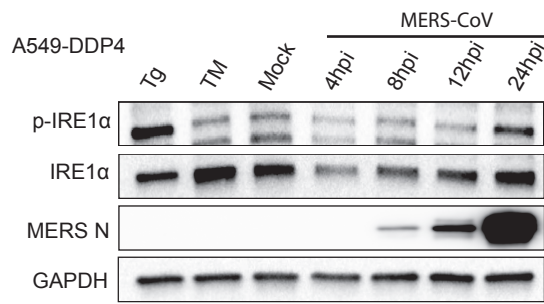**B**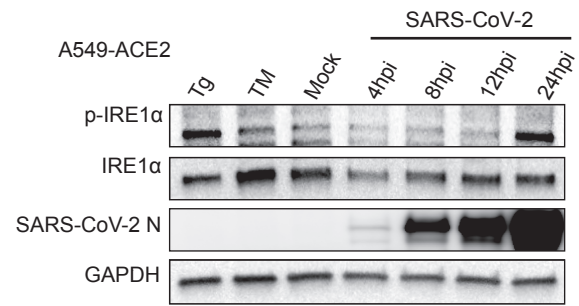

Supplement: FIG S1 [file mbio.02415-22-s0001.pdf]

**A**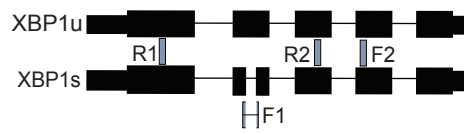

$$\% \text{ XBP1s} = \frac{\text{XBP1s(R1, F1)}}{\text{XBP1total(R2, F2)}}$$

**B**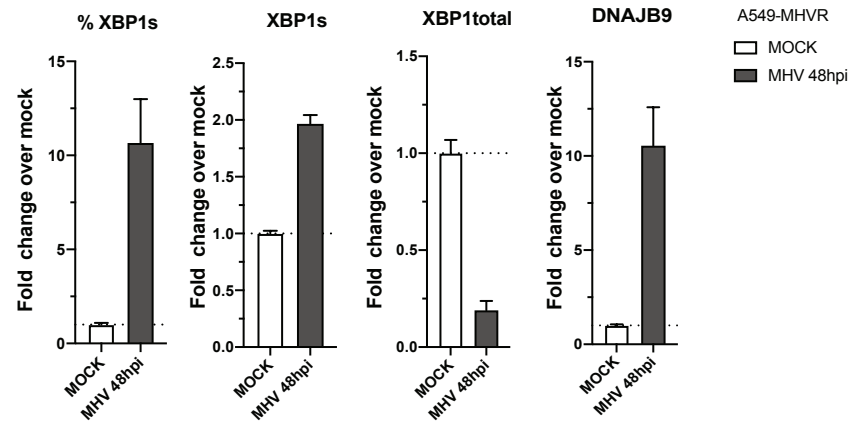

Supplement: FIG S2 [file mbio.02415-22-s0002.pdf]

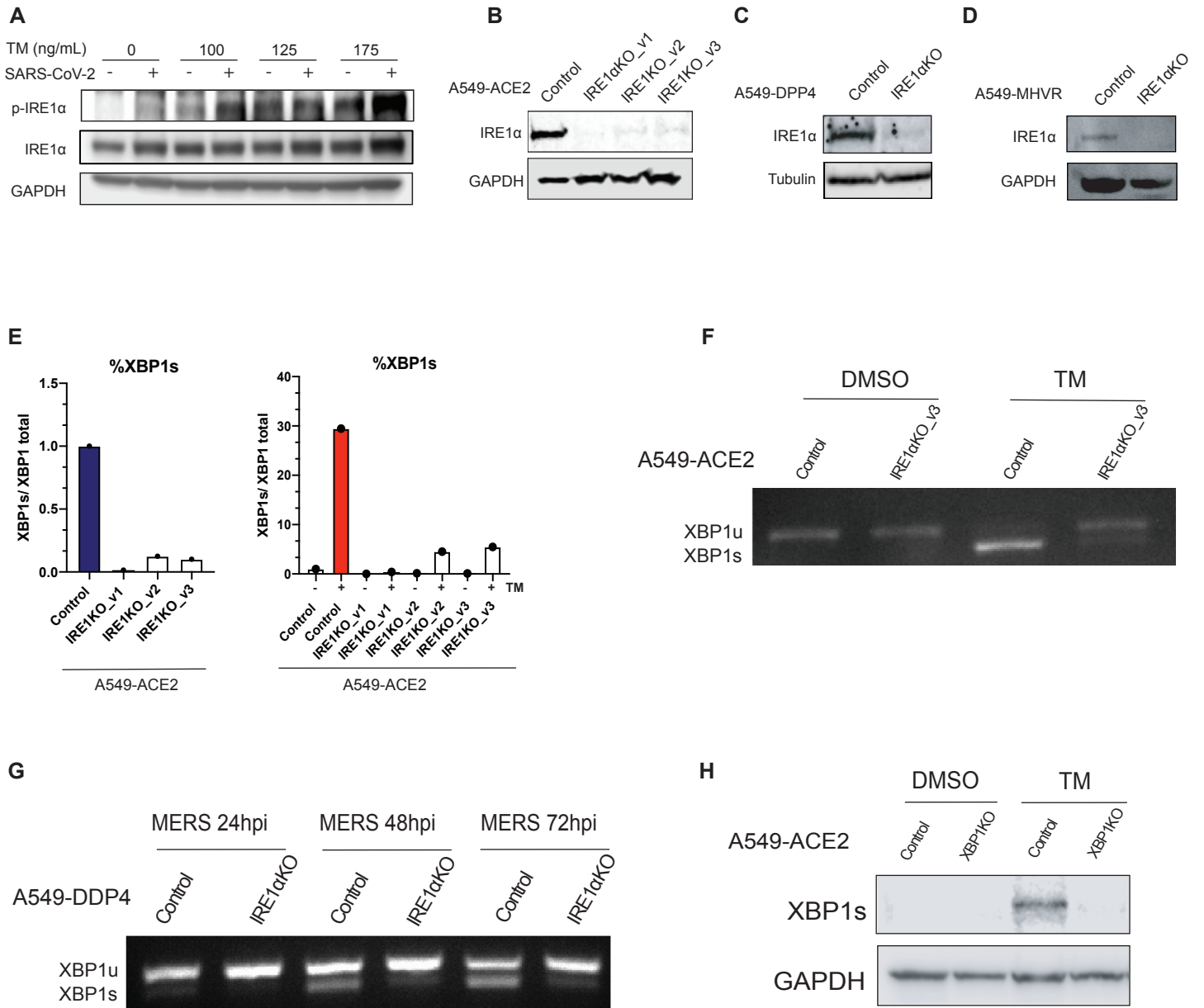

Supplement: FIG S3 [file mbio.02415-22-s0003.pdf]

A

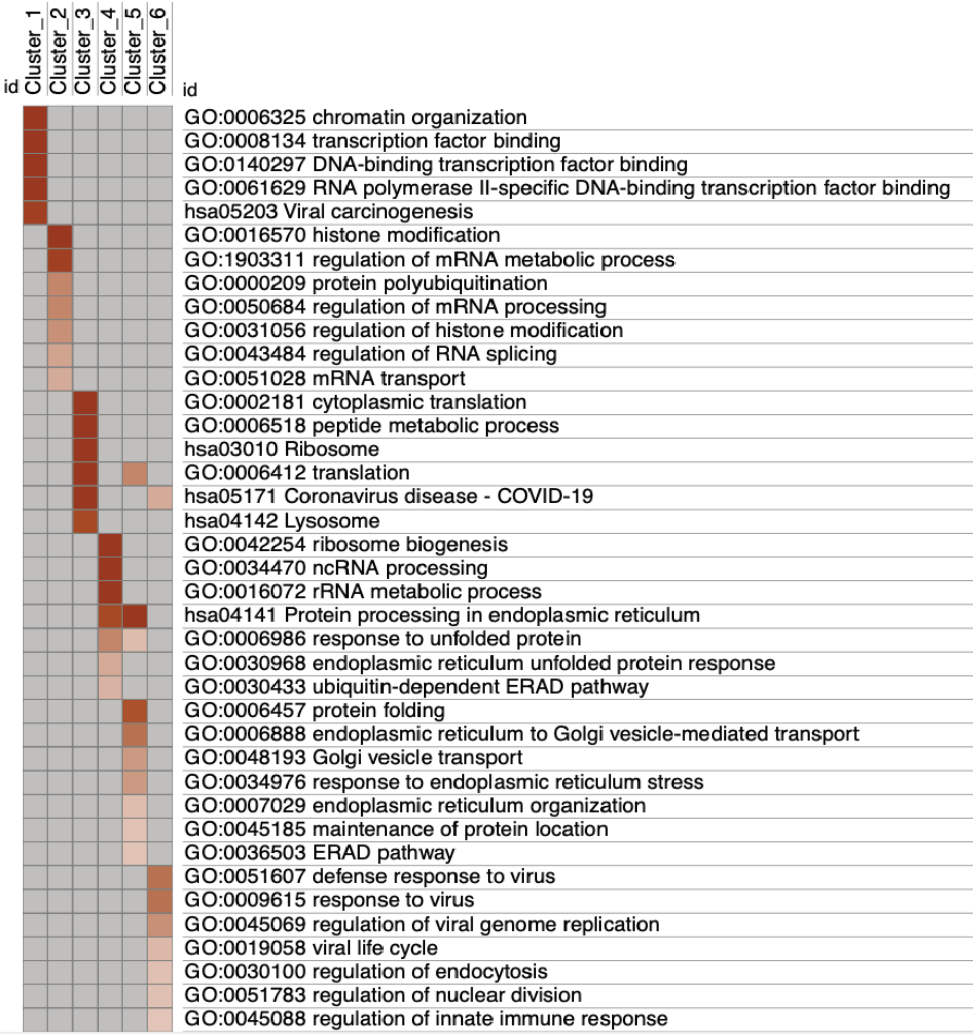

B

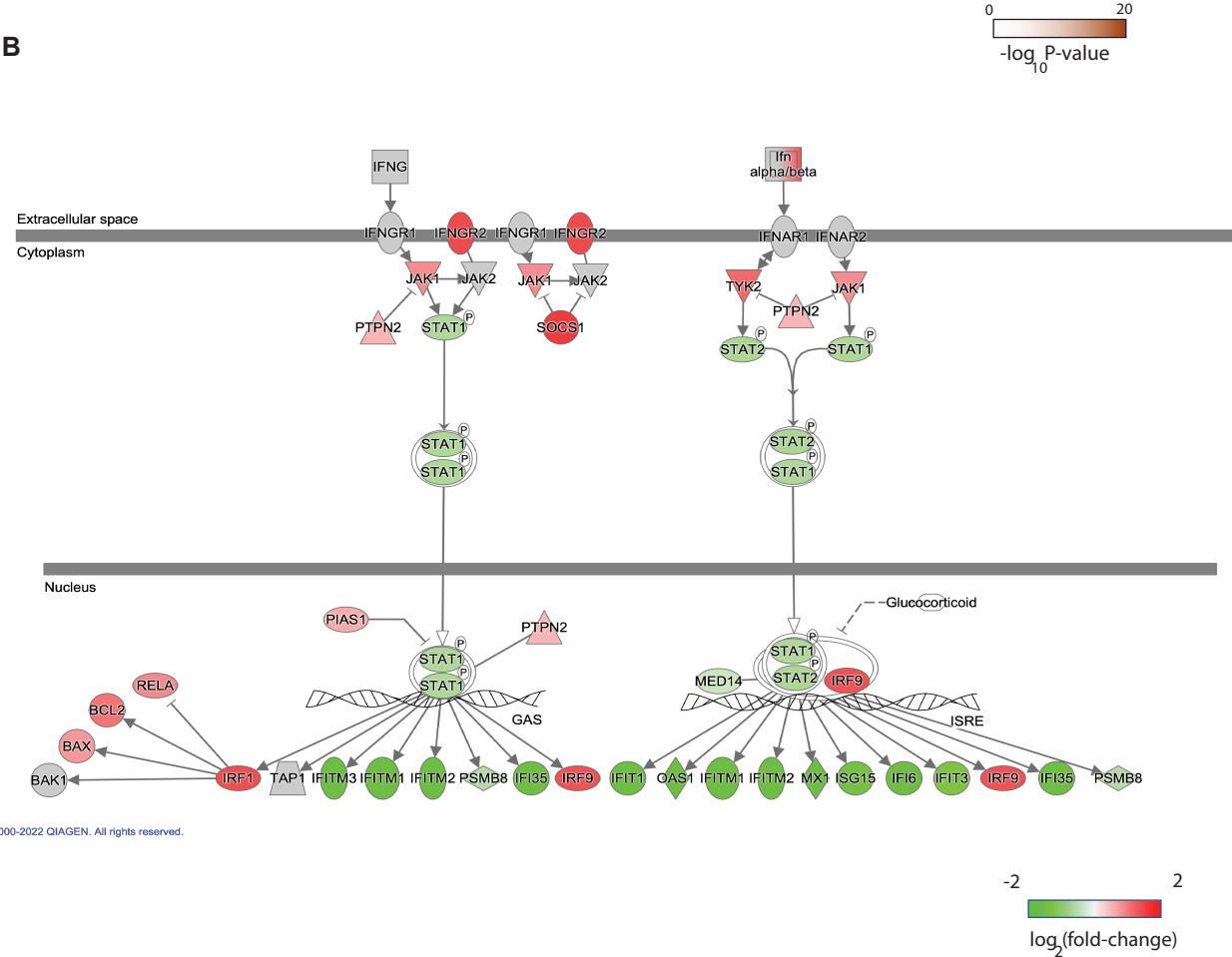

Supplement: FIG S5 [file mbio.02415-22-s0005.pdf]

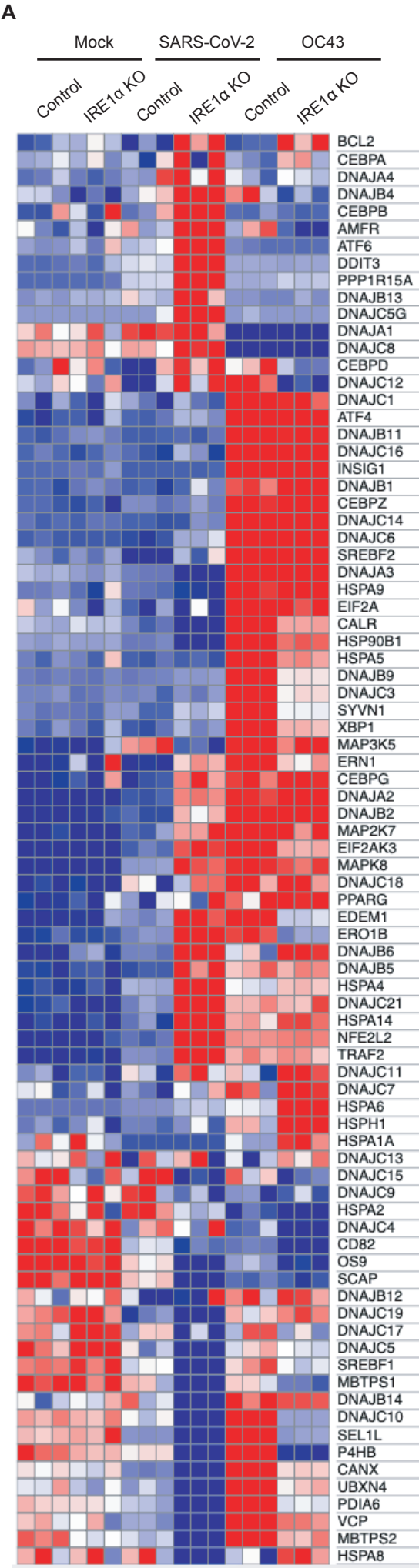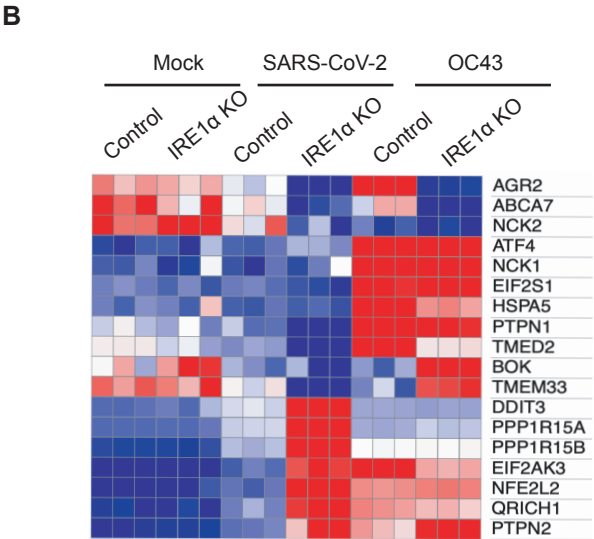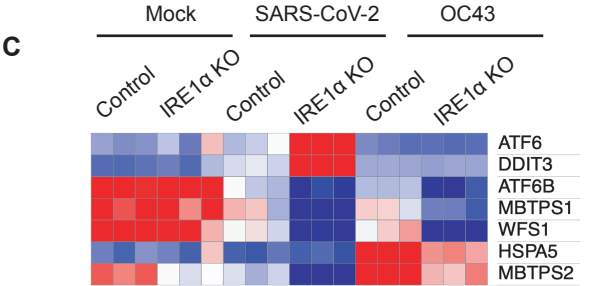

Supplement: FIG S6 [file mbio.02415-22-s0006.pdf]
