# Supplementary material for: SARS-CoV-2 Diverges from Other Betacoronaviruses in Only Partially Activating the IRE1α/XBP1 Endoplasmic Reticulum Stress Pathway in Human Lung-Derived Cells
Source: mBio. 2022 Sep 20;13(5):e02415-22. doi: 10.1128/mbio.02415-22 (PMC9600248; doi:10.1128/mbio.02415-22)

Supplement Figure 4

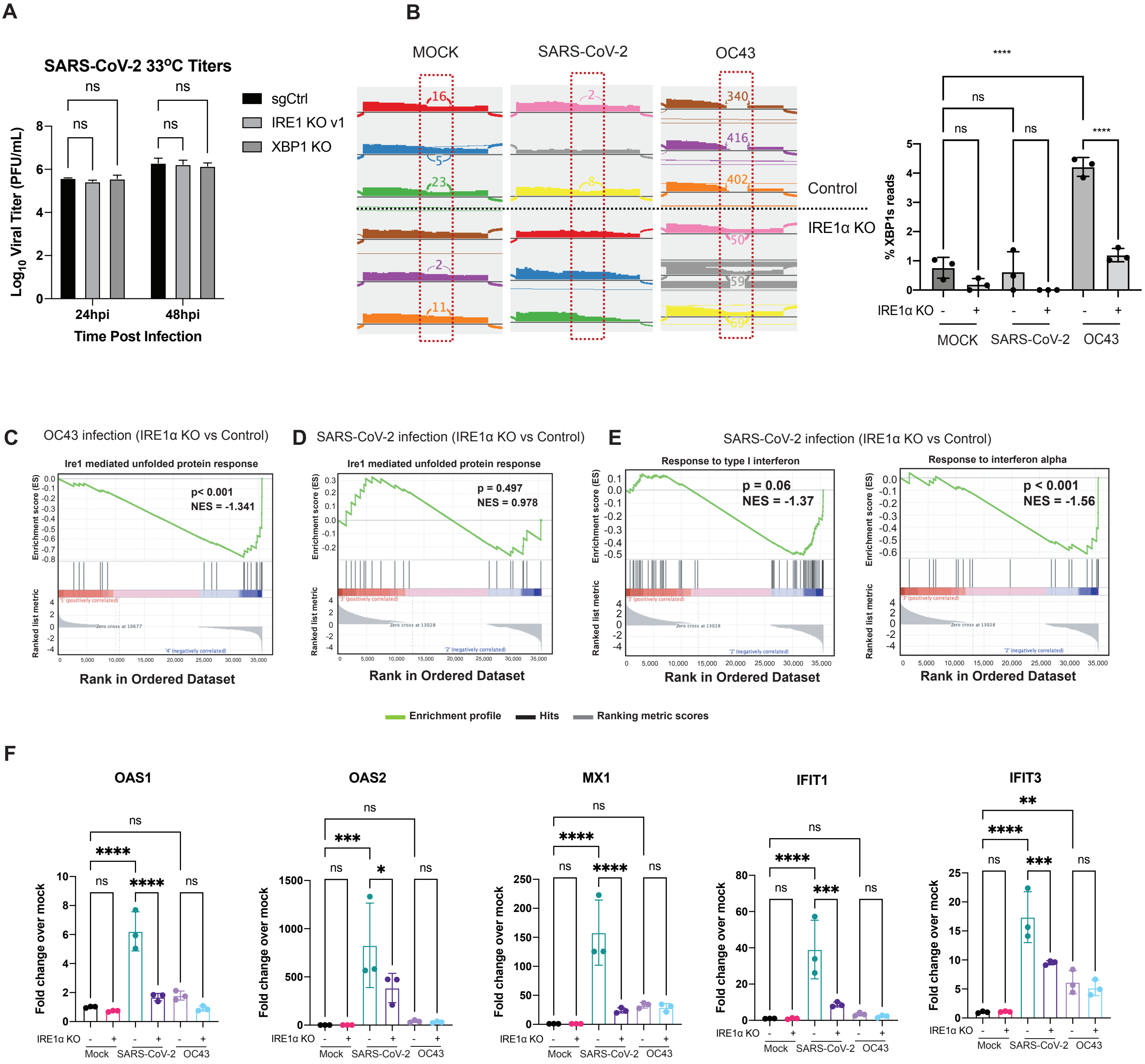

Supplement: FIG S4 [file mbio.02415-22-s0004.pdf]
